# Development of Cross-Reactive Antibodies for the Identification and Treatment of Synthetic Cannabinoid Receptor Agonist Toxicity

**DOI:** 10.3390/vaccines10081253

**Published:** 2022-08-04

**Authors:** Adam Worob, Cody J. Wenthur

**Affiliations:** Pharmaceutical Sciences Division, School of Pharmacy, University of Wisconsin-Madison, Madison, WI 53705, USA

**Keywords:** cannabinoids, antibodies, behavior, cross-reactivity, chemoinformatics

## Abstract

Synthetic cannabinoid receptor agonists (SCRAs) are compounds that mimic the pharmacology of the psychoactive components in cannabis. These compounds are structurally diverse, inexpensive, commercially available, and difficult to identify with modern analytical methods, making them highly accessible for recreational use. Suspected SCRA toxicity, which can present with a breadth of cardiovascular, gastrointestinal, and neurological disturbances, is currently addressed through symptom management followed by a toxicological screening that often occurs long after patient discharge. Here, we report the development of four cross-reactive anti-SCRA bioconjugate vaccines as a platform for developing improved diagnostic and therapeutic interventions against SCRA intoxication, using SCRA-resembling small molecule haptens that combine common subregional motifs occurring within and across different generations of SCRA molecules. Using a combination of multiplexed competitive ELISA screening and chemoinformatic analyses, it was found that the antibodies resulting from vaccination with these bioconjugates demonstrated their ability to detect multiple SCRAs with a Tanimoto minimum common structure score of 0.6 or greater, at concentrations below 8 ng/mL. The scope of SCRAs detectable using these haptens was found to include both bioisosteric and non-bioisosteric variants within the core and tail subregions, as well as SCRAs bearing valine-like head subregions, which are not addressed by commercially available ELISA screening approaches. Vaccination with these bioconjugates was also found to prevent the changes in locomotion and body temperature that were induced by a panel of SCRAs at doses of 1 and 3 mg/kg. Further refinement of this genericized hapten design and cross-reactivity-prioritizing approach may enable the rapid detection of otherwise cryptic SCRAs that arise during overdose outbreaks, and could ultimately lead to identification of monoclonal antibody species applicable for overdose reversal.

## 1. Introduction

Synthetic variants of drugs which elicit psychotropic effects through central nervous system mechanisms are known as novel psychoactive substances (NPS). Several common categories of NPS have been identified historically, including opioid and serotonin receptor agonists, with more recent expansion to include cannabinoid 1 receptor (CB1R) agonists, as well [1,2,3]. Despite their relative nascence, synthetic cannabinoid receptor agonists (SCRA) represent the most rapidly proliferating class of NPS, with over 260 compounds identified to date [4]. These compounds mimic the psychotropic effects of biosynthetic cannabinoids, leading to slowed cognition, euphoria, and altered vigilance. In contrast to delta-9-tetrahydrocannabinol (Δ^9^-THC), which is the major psychoactive phytocannabinoid in cannabis, and a partial CB1R agonist, most known SCRAs are full CB1R agonists, leading to a unique toxicological profile for these compounds [5]. For example, SCRA consumption has been associated with cardiotoxicity, severe psychosis, and even death in some cases, while such effects are rarely observed following cannabis ingestion [6,7].

Despite the serious health concerns associated with SCRA use, detection methods for SCRAs in blood, urine, or saliva from patients with suspected ingestion remain scarce and largely impractical for clinical screening. One of the most common methods for the detection and identification of cryptic illicit NPS is liquid chromatography coupled to mass spectrometry (LC-MS), and many advances have been made to improve the detection capacity of LC-MS for individual SCRA structures, as well as mixtures of several SCRAs and related metabolites, with high accuracy [8,9,10,11,12]. However, despite these impressive technical and analytical developments, high-resolution mass spectrometers remain expensive, and they are not yet routinely available in clinical lab settings; expert opinion suggests that despite improved uptake, permanent educational support and improvements in automation are critical challenges to address if mass spectrometry approaches are to become a routine part of clinical chemistry operations over the next 10–20 years [13]. Survey data of hospital laboratories support the challenges for widespread implementation of LC-MS approaches for this use, revealing that even amongst users that had adopted LC-MS assays, fifty percent of respondents had switched from LC-MS testing back to another platform, with most of these respondents noting that complexity and workload were part of the rationale for this decision. Finally, in real world cases using LC-MS approaches for SCRA-sample analysis, high resolution LC-MS confirmation of SCRA presence in biological samples can take up to 10 days, a time scale more than 25 times longer than reported for symptom resolution in the outbreak index case (9 h) [14].

These pragmatic considerations, which are likely to be amplified in resource-poor environments, provide a strong rationale for continuing innovation of immunologic, enzyme-linked immunosorbent assay (ELISA) approaches as a cheaper, more accessible method for real-time SCRA detection. Furthermore, the development of antibody-based approaches for screening has a unique advantage that cannot be matched by LC-MS techniques—their potential to be further leveraged as therapeutic interventions via monoclonal infusion for overdose reversal. Unfortunately, to date, SCRA immunoassay performance has been insufficient to meet the standards required of clinical applications for either detection or therapeutic intervention. A combination of two commercial SCRA ELISA kits yielded only 2% sensitivity, and 51% diagnostic accuracy at the recommended cutoff limits of 20 ng/mL. Altogether, these tests performed only slightly above chance levels; an especially notable reason for this poor performance is insufficient cross-reactivity of these assays for the detection of newer generations of SCRAs [15].

**Figure 1:**
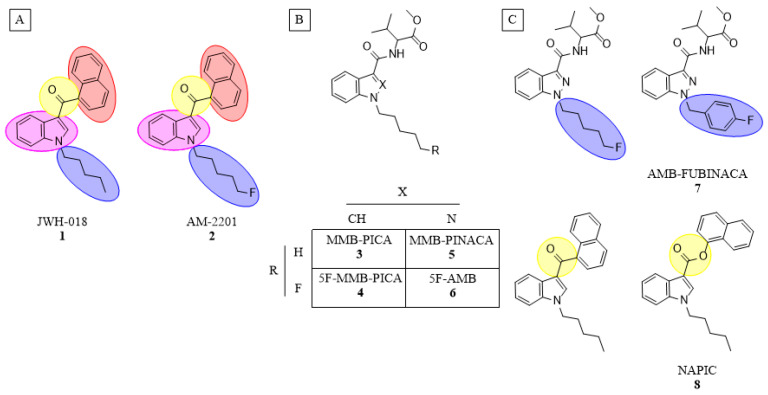
Relevant SCRA species for this study. (**A**) General SCRA structure containing a head (red), linker (yellow), core (pink) and tail (blue) subregion. (**B**) Valine-like head SCRAs detailing minor changes within an SCRA class. (**C**) Other common structural variations in tail and core subregions.

As with earlier SCRAs, these new compounds can be structurally conceptualized as having four major subregions (Figure 1A). These subregions include an often aromatic and easily functionalized core, a highly variable head, a linker connecting these two, as well as an alkyl tail. While relatively subtle structural modifications to the core and linker subregions have occurred in newer generations of SCRA compounds, the incorporation of valine- or tert-leucine (i.e., non-aromatic) species in the head subregion and the inclusion of a wider variety of tail structures were particularly notable alterations to SCRA molecules that led to detection failure by the commercial SCRA ELISA kits. Given that the ease ofSCRA structural modification is a major contributor to the generation of novel SCRA analogues, a successful immunodiagnostic approach must be able to recognize both multiple generations of SCRA structures, as well as multiple structurally similar SCRAs within a given generation. Historically, the creation of high-fidelity antibodies directed against a single target structure has been employed successfully for the detection of heroin, prescription and synthetic opioids, nicotine, methamphetamine and various NPS compounds; only recently have there been dedicated efforts to create promiscuous antibodies that recognize multiple structural analogues within a target class of NPS [16,17,18,19,20,21,22].

Our exploration into the creation of a broader spectrum of cross-reactive anti-SCRA antibodies begun with the synthesis of four haptens in an attempt to assess both the major structural differences that can exist within the head subregion, as well as the more subtle modifications common within the core and linker subregions. Upon bioconjugation and vaccine formulation, the biological relevance of inoculation was assessed by the administration of structurally related SCRAs, which elicits a reliable pattern of quantifiable physiological changes. Finally, antibody-containing sera were tested in vitro for reactivity against SCRAs with increasing structural diversity to assess cross-reactivity.

## 2. Materials and Methods

**Chemical synthesis:** Detailed methods for all hapten and SCRA syntheses can be found in the supplemental information. ^1^H and ^13^C nuclear magnetic resonance (NMR) spectra were obtained using a Bruker Avance III HD 400 MHz NMR Spectrometer and analyzed with MNova. Chemical shifts are reported in ppm from internal CDCl_3_ (^1^H—7.26, ^13^C—77) and DMSO-d_6_ (^1^H—2.50, ^13^C—39.51) solvent standards.

**Bioconjugation:** Haptens were dissolved in a 10% DMSO in 1X PBS solution (2 mg/mL) and activated at room temperature with Ethyl-3-(3-dimethylaminopropyl)carbodiimide (EDC) and sulfo N-hydroxysuccinimide (sNHS). Equal amounts of 2 mg/mL Bovine serum albumin (BSA) or Keyhole Limpet Hemacyanin (KLH) dissolved in 1X PBS after hapten consumption was visualized via thin layer chromatography (TLC). The protein activation continued overnight at 4 °C before the bioconjugates were purified using Pierce Zeba Desalt spin columns. Copy numbers for each BSA-bioconjugate was determined using matrix-assisted laser desorption/ionization—time of flight (MALDI-ToF) analysis and the following equation: Copy # = (MW_BSA-HAPTEN_—MW_BSA_)/(MW_HAPTEN_—18.02).

**Animals:** Research animals for this study consisted of 6- to 8-week-old male Swiss–Webster mice with ad libitum access to food and water. Animals were housed in an American Association for Accreditation of Laboratory Animal Care (AAALAC) accredited vivarium with a reverse light cycle. All experimental protocols adhere to the University of Wisconsin Institutional Animal Care and Use Committee (IACUC) and the National Institute of Health Guide for the Care and Use of Laboratory Animals.

**Vaccination:** Vaccine formulation consisted of our bioconjugates at 1 mg/mL (100 μg/mouse), Alum (50 μg/mouse) and a 1 mg/mL CpG ODN 1826 (50 μg/mouse) in 1X PBS solution. Vaccines were shaken at 4 °C for 45 min prior to animal use. Mock vaccines consisted of the same components with the bioconjugate replaced with native KLH. All animals were vaccinated on day zero and received two boosts, one on day 14 and one on day 28. Sera was collected from each animal on days 21 and 35 via retroorbital blood draws.

**Cannabinoid Tetrad:** All animals underwent the cannabinoid tetrad protocol on days 37 (3 mg/kg) and 44 (1 mg/kg) following IP injection of the SCRA with the highest chemical similarity to the haptenic structure. Vaccine groups 1–4 received **1**, **8**, **3** and **5**, respectively, while mock vaccinated animals received **7**. After 25 min the animals were put into an open field apparatus and their overall locomotion was monitored over 15 min using ANY-Maze tracking software. Within 1–2 min after the open field test, the core temperature of the animals was ascertained with a rectal thermometer. Nociception was then measured using a hot-plate set to 52 °C with a cut-off time of 30 s to avoid any injury to the animals. Lasty, catalepsy was measured by placing the front paws of the mouse on an elevated bar and determining the latency to remove both paws. Cut-off time for each trial was 180 s and the results from three trials were averaged. Two-way ANOVAs were used to assess main effects of dose, main effects of vaccination, and dose x vaccine interactions for each of the tested SCRAs.

**Antibody Titers:** Costar^®^ 96-well plates were coated with 25 μL of a 5 μg/mL solution of hapten-BSA conjugate and incubated overnight at 37 °C. Plates were then methanol fixed and non-specific protein binding was blocked with 5% Blotto for 30 min. Pooled sera from either day 21 (1:100 in 5% blotto) or 35 (1:400) were added and serially diluted across the plate before incubating at 37 °C for 60 min. Plates were then washed with 1X PBS prior to the addition of secondary antibody conjugated to horse-radish peroxidase, which incubated at 37 °C for 30 min. Equal parts tetramethylbenzidine and hydrogen peroxide (50 μL) were added to each well and reacted for 15 min before being quenched with 50 μL of 2M sulfuric acid. Optical densities were determined at 450 nm using MiniMax 300 plate reader (Molecular Devices). Lastly, midpoint titers were calculated from the % optical density versus log(dilution) using the log(inhibition) vs response variable slope equation on GraphPad Prism v9 (San Diego, CA, USA).

**Competitive ELISA:** Costar^®^ 96-well plates were coated as described in the antibody titer section. After the plates are blocked, 25 μL of a 1:titer solution of each BSA-hapten conjugate is added to every well. Equal volume of a 40 μM solution of each SCRA is then added to column one, and 1:2 serially diluted concentrations added across the wells. Optical densities were determined as in titer experiments, and percent inhibitions were evaluated with GraphPad Prism 9 as in titer experiments.

## 3. Results

While substantial chemical and structural differences exist among the full spectrum of known SCRAs due to multiple parallel avenues of modification being attempted over time, subtle chemical modifications to known SCRAs with modest impacts on pharmacodynamic properties are a common source leading to the emergence of new compounds within a given generation. Indeed, structural differences among SCRAs within a generation are often bioisosteric, producing similar biological effects to one another despite varying electronic properties (Figure 1B). Common examples include replacing indole cores with indazoles or the introduction of a fluorine to the terminal carbon of pentyl tails [23,24,25,26,27]. With this context in mind, a panel of eight different SCRAs was selected for study, and demarcated into two generations based on differences in the head subregion (i.e., Naphthalene (**1**, **2** and **8**) and valine methyl ester (**3**–**7**); Figure 1). The extent to which facile structural changes between molecules influence antibody recognition of small-molecule targets is largely unknown, and the rapid evolution of SCRAs compounded with the unpredictable nature of overdose outbreaks makes generating new haptens for each individual new compound unrealistic. Therefore, anti-SCRA hapten designs should take advantage of these structural similarities within SCRA generations to allow for maximal antibody cross-reactivity, a property which enables the recognition of multiple compounds with minor alterations from the haptenic structure [28,29,30,31].

**Scheme 1:**
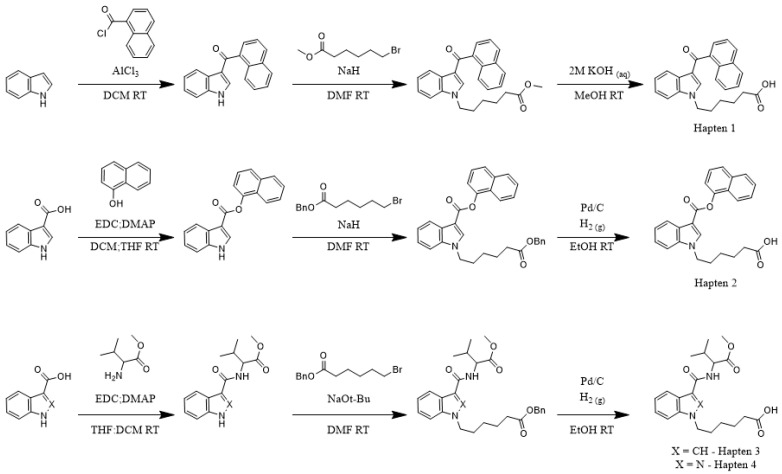
Syntheses of the four different haptens exploring differences within the head, linker and core subregions.

To determine the correlation between hapten and target molecule similarity and resulting antibody affinity, we designed four haptens which mirror some of the minor and major changes observed both across and within different generations of commonly encountered SCRAs (Scheme 1). Haptens 1 and 2 are identical aside from the linker subregion, and haptens 3 and 4 are identical aside from their core subregion. Between these hapten pairs, however, large, non-bioisosteric changes to the head and linker are apparent, reducing the probability of cross-reactivity between generations. In addition, all four haptens are appended with a hexanoic acid tail, with the acid representing an activatable functional group for bioconjugation to carrier proteins, which we denote as the bioconjugation site. This site was chosen to maximize the presentation of core, linker and head subregions of the SCRA-resembling haptens to the immune system, a concept known as facial selectivity, which was originally detailed for anti-heroin vaccines [29]. Furthermore, as vast structural diversity is observed within the head subregion of known SCRAs, we hypothesized that maximizing presentation of the SCRA head would generate higher affinity antibodies for each generation of SCRAs under study, as compared to hapten designs prioritizing presentation of less structurally variable subregions.

**Figure 2:**
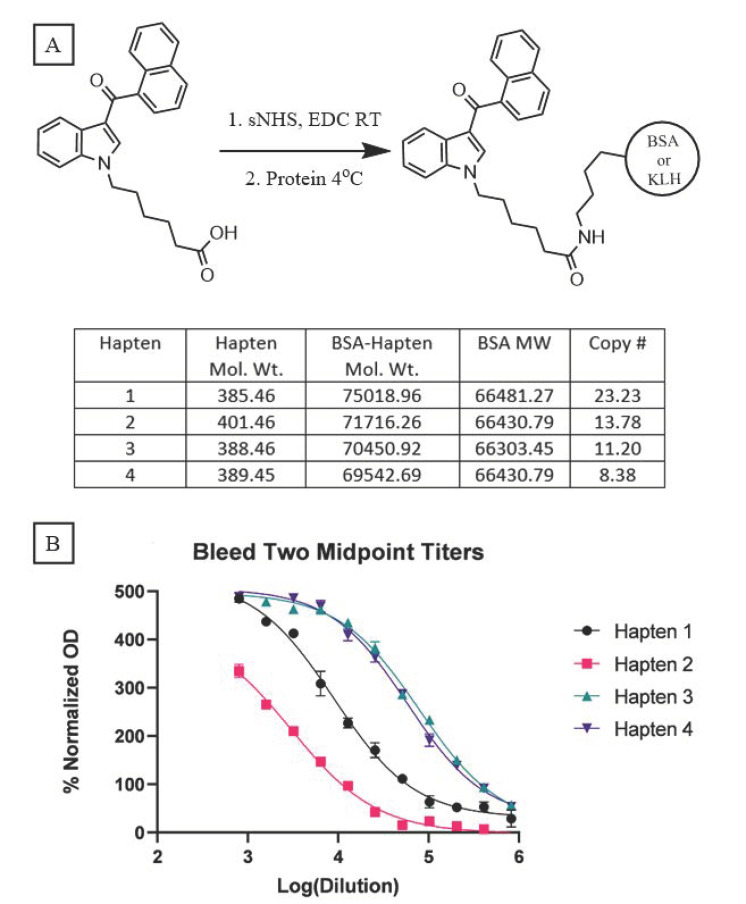
(**A**) Bioconjugation conditions and copy numbers for each hapten. (**B**) Antibody titers from pooled sera collected on day 35. Data shown as mean ± SEM.

Because our haptens do not activate the immune system when administered alone, conjugation to an immunogenic carrier is necessary to generate anti-SCRA antibodies. These reactions proceeded with standard bioconjugation conditions (Figure 2A) prior to the addition of either Keyhole Limpet Hemocyanin (KLH) or Bovine Serum Albumin (BSA). While KLH is inexpensive and quite immunogenic, its large size makes copy number analysis impractical [32]. In contrast, BSA is a less immunogenic, but well characterized, carrier, permitting routine copy number analysis. Furthermore, BSA bioconjugates also serve as orthogonal probes during ELISA, mitigating binding of antibodies with paratopes directed at the KLH carrier rather than the hapten. Using identical reaction conditions for the conjugation of all four haptens, MALDI-ToF analysis of the BSA bioconjugates (Supplementary Figures S1–S4) revealed a range of copy numbers with a difference of 15 copies between the highest and lowest-density bioconjugates (Figure 2A). All vaccines were formulated with Alum and CpG ODN 1826, and the resulting vaccines were administered to mice following a protocol of vaccination on days 0, 14, and 28 and sera collection on days 21 and 35.

Sera collected from vaccinated mice were pooled for each hapten and control group (n = 10), and analyzed by an indirect ELISA to determine the antibody titers. Blood from either day was pooled within each group, with the day 35 titers showing large differences between haptens 1 and 2 versus 3 and 4 (Figure 2B); vaccines from the former two elicited considerably weaker immune responses compared to the latter two. A comparable trend is observed with the pooled sera from day 21 (Supplementary Figure S5). Hapten 2, the least immunogenic of the four, warrants special consideration as its ester linker is more chemically labile compared to the amide and ketone linkers. Hydrolysis of this naphthyl ester, either during bioconjugation or following vaccination, can cause the loss of the head subregion resulting in the presentation of a different molecule to the immune system, as recently reported for similar ester-bearing haptens in vivo [33]. Consequently, antibodies generated against this vaccine may lack the ability to bind the cognate molecule **8**. Despite an almost inverse relationship existing between conjugation numbers and antibody titers for our haptens, a clear direct relationship is observed between titer and vaccine performance.

Since our vaccines generated significant titers of antibodies against SCRA-mimicking haptens, we further evaluated their in vitro affinity for specific SCRAs with increasingly distinct structures from the hapten design. For each of the four vaccine conditions, the sera from day 35 was thus assessed in a competitive ELISA assay to evaluate antibody recognition of each of the eight individual SCRAs in our testing panel. Table 1 provides the percent inhibition values against each tested SCRA at 10 μM, along with the IC_50_ values for antibody inhibition of binding, as determined across a range of 11 concentrations (Supplementary Figure S6). Antibodies generated from vaccination with hapten 1 were able to inhibit binding of JWH-018 (**1**) and the 5-fluoro analog AM-2201 (**2**) at micromolar concentrations of drug. The IC_50_ value for hapten 1 against the fluorinated compound **2** was approximately five times lower than against compound **1,** despite the lack of fluorine in the hapten design. It is also worth noting that hapten 1 antibodies showed no reactivity towards NAPIC (**8**) despite the only structural difference being that **8** has an ester instead of an acyl linkage. Hapten 2, which elicited the weakest immune response of all the haptens, showed little to no reactivity towards our SCRA panel, even against its cognate compound **8**. As haptens 3 and 4 only differed by a single heteroatom in the core subregion, we expected there to be considerable cross-reactivity between the indole and indazole compounds. Indeed, antibodies generated following vaccination with hapten 3 were able to bind MMB-PICA (**3**) and its 5-fluoro analog (**4**), as well as the indazole core SCRAs MMB-PINACA (**5**) and the fluorinated analog 5F-AMB (**6**) at micromolar to sub-micromolar concentrations. The same pattern of cross-reactivity was observed following vaccination with the indazole containing hapten 4, generating antibodies that bind SCRAs with both indazole (**5** and **6**) and indole (**3** and **4**) cores. In contrast to hapten 1, however, haptens 3 and 4 both preferentially bound to the non-fluorinated analogs. In addition, antibodies created against both haptens 3 and 4 were also inhibited by AMB-FUBINACA (**7**), an SCRA with a structurally non-bioisosteric tail, at sub-micromolar concentrations.

**Figure 3:**
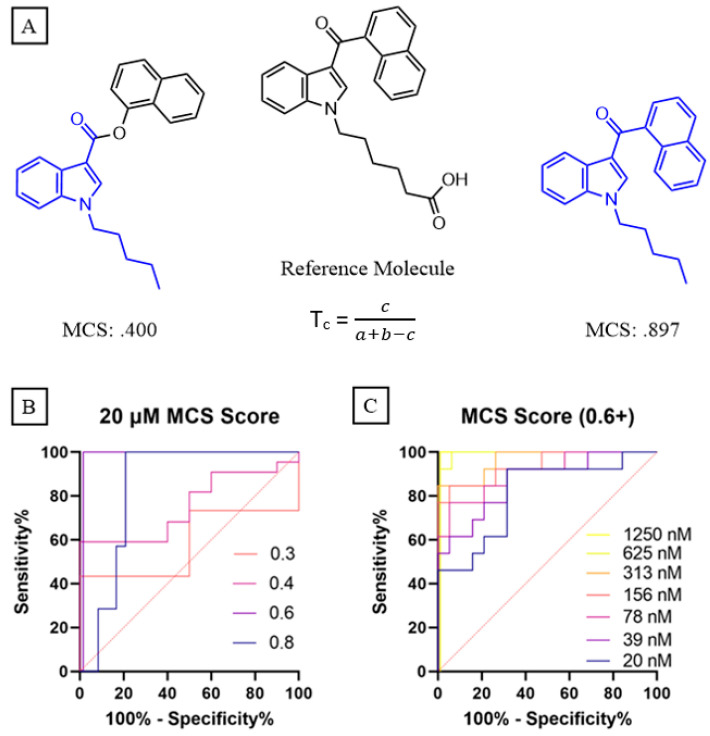
(**A**) Maximum common substructure (MCS) Tanimoto coefficients calculated for two structurally similar SCRAs to hapten 1 (reference molecule), where c equals the number of common non-hydrogen atoms between the molecules, and a and b equal the total number of non-hydrogen atoms for either compound. (**B**) Receiver operating characteristic (ROC) analysis at 20 μM dilutions, showing perfect discrimination (AUC = 1) for SCRA-hapten pairs with an MCS Tanimoto coefficient of 0.6 or greater. (**C**) ROC analysis for all competitive ELISA dilutions based on the 0.6 MCS threshold determined in 3b.

As research into the use of antibodies for small molecule detection continues to grow, so does the availability of data that relates hapten design to downstream antibody performance. This relationship becomes especially important when considering the breadth of known SCRAs and their propensity to evolve into novel compounds with facile chemical changes. Therefore, we next wanted to leverage these competitive ELISA data to pilot an approach that quantifies the ability of these anti-SCRA antibodies to bind to compounds occupying a specific region of chemical space, including presently unidentified analogues that may arise from within this space. To begin this process, we started with two well-established cheminformatic metrics for assessing compound similarity: atom pair (AP) and maximum common substructure (MCS) Tanimoto scores (Figure 3A) [12,34,35]. These AP and MCS scores were generated for every SCRA-hapten combination (Supplementary Table S1) using the online ChemMine Tools platform and plotted against two different measures of antibody effectiveness, IC_50_ and % inhibition [36]. This structural similarity analysis was performed for all 32 SCRA-hapten pairs within this study, as well as for recent anti-SCRA antibody data generated by Lin et al. (Supplementary Figure S7). Whereas the data from this research present a clear bimodal distribution of SCRA-hapten pairs, a much broader distribution is present in the larger Lin et al. data set. In both cases, the expected positive relationship is apparent between these SCRA-hapten similarity scores and the ability of the resulting antibodies to recognize the relevant SCRA.

This promising initial pattern encouraged us to evaluate the predictive power of the structure-performance relationships generated for each SCRA-hapten pair in our study through a receiver operating characteristic (ROC) analysis. To define an initial threshold for what degree of chemical similarity could be detected through reliance on antibody cross-reactivity, we analyzed the performance of the antibodies across various MSC and AP Tanimoto cutoff scores for structural relatedness at the maximum SCRA concentration (20 μM). Under these conditions, the MCS metric performed better than AP overall. Notably, the MCS score ‘cross-reactivity’ threshold of 0.6 generated the theoretical maximum area under the curve (AUC) of 1, indicating perfect discriminatory capability between SCRAs that share 75% or more of their substructure with a test hapten versus those that do not, as calculated using the MCS approach (Figure 3B) [34].

Given that 20 μM concentrations are well in excess of those seen for clinical cases of SCRA intoxication, the diagnostic fidelity of this selected similarity threshold was next assessed at sequentially decreasing SCRA concentrations. Perfect discrimination for the 0.6 MSC threshold was observed in our dataset for all concentrations above 0.625 μM. Even at the lowest tested concentration of 20 nM, the AUC for cross-reactivity detection was 0.81 (Figure 3C), which compares favorably to commercially available approaches regarding both discriminatory performance (AUC: 0.51–0.56) and detection cutoff (10–20 ng/mL). At this lowest dilution for the eight SCRAs in this study (average MW = 357.19), this means an approximate concentration of 7.14 ng/mL can be detected for all tested compounds that exceed the 0.6 MCS similarity threshold. Both fatal and non-fatal cases for intoxication with JWH-018, AM2201, and AMB-FUBINACA have been reported to yield blood concentrations above this detection limit; there have also been several high-profile cases in which verified consumption of an SCRA has led to blood concentrations in the hundreds of ng/mL, such as the ‘zombie’ outbreaks associated with AMB-FUBINACA, indicating that this cutoff limit is relevant for detection in serious and life-threatening cases [14,37,38] However, further performance improvements are likely needed for broad utility, as in many cases of SCRA intoxication with unregulated material, blood concentrations are often sub-nanomolar [37].

**Figure 4:**
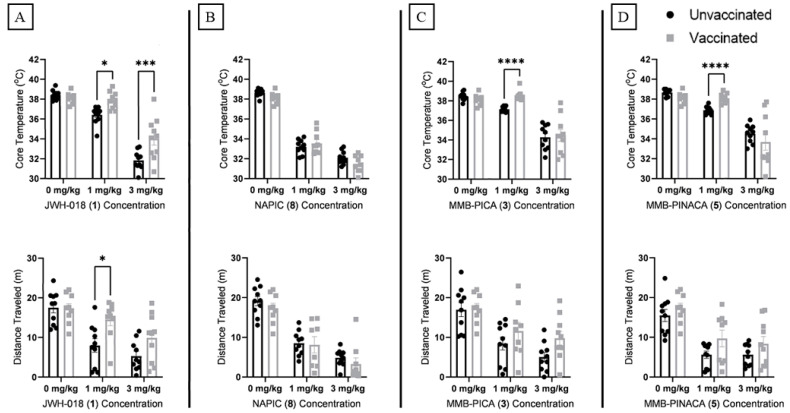
Core temperature and locomotion data for vaccine groups 1 through 4 ((**A**–**D**), respectively). Results were analyzed by a two-way ANOVA with Šidák multiple comparison correction. *p* values: 0.05 (*), 0.001 (***) and 0.0001 (****). Data shown as Mean ± SEM.

Next, since reversal of overdose is a unique potential benefit of antibody-based technologies, in comparison with LC-MS approaches, in vivo vaccine efficacy was also assessed in the presence of 1 and 3 mg/kg doses of SCRAs using the cannabinoid tetrad battery, which measures four physiological or behavioral responses indicative of cannabinoid intoxication [39,40]. The SCRAs administered were those most structurally similar to the hapten design, resulting in vaccine groups 1–4 receiving compounds **1**, **8**, **3** and **5**, respectively. Twenty-five minutes after administration, the animals were assessed for reductions in overall locomotion and core body temperatures (Figure 4) as well as reductions in nociception and induction of catalepsy(Supplementary Figure S8) with Two-Way ANOVAs. Following a saline injection, no main effects on locomotion or temperature were observed due to vaccination alone. When considering the main effect of drug administration in unvaccinated animals, all four SCRAs induced significant hypothermia and hypolocomotion at doses of 1 or 3 mg/kg, validating that these compounds are cannabinoid-like in nature. Interactions between SCRA dose and vaccination status were observed, as immunization with hapten 1 caused significant rescue of both hypolocomotion and hypothermia induced by 1 mg/kg of JWH-018 (**1**). Immunization with haptens 3 and 4 also elicited significant rescue of MMB-PICA (**3**) and MMB-PINACA (**5**) induced hypothermia, respectively. Immunization with hapten 2 showed no ability to rescue hypothermia or hypolocomotion induced by NAPIC (**8**) at either drug concentration. This observation, in tandem with the capacity of **8** to induce physiological alterations in mice indicative of cannabinoid receptor activation, is consistent with the interpretation that structures like hapten 2 are subjected to degradation during the vaccination process [33]. While varying levels of physiological rescue were seen at 1 mg/kg doses, 3 mg/kg doses for all four SCRAs elicited considerable decreases in overall locomotion and core temperature, and the influence of the vaccinations was largely overcome. A notable exception to this trend was the ability of hapten 1 to significantly reverse hypothermia induced by JWH-018 (**1**), even at this elevated 3 mg/kg dose. The otherwise limited differences between vaccination groups at this elevated dose suggests saturation of sera antibodies with the remaining free drug being sufficient to elicit significant physiological effects. No significant interactions arising from vaccination were seen at either drug concentration for the hotplate and catalepsy tests in all four vaccination groups, due in part to high individual variability in SCRA effects on these measures. Nevertheless, the ability of our chemically stable hapten vaccines to demonstrate notable reversal of SCRA-induced locomotion and body temperature deficits at 1 mg/kg, and up to 3 mg/kg in some cases, suggests promise for future optimization of high-affinity anti-SCRA monoclonal antibodies as overdose reversal agents.

## 4. Discussion

With these findings, we can identify several areas for future study and technological improvement. The poor performance of hapten 2 demonstrates the importance of prioritizing hapten stability, as hydrolysis of naphthyl esters in mouse sera has been observed within days; indole-3-carboxylate thus likely served as the presented antigen for a majority of the vaccination schedule with this hapten. Future syntheses of bioisosteric haptens with metabolically stable amide or acyl-methylene linkers should be undertaken to develop antibodies capable of recognizing related diaryl ester/amide SCRAs.

The bioconjugation approach for SCRAs presents another area for further optimization, which may involve methods to control rather than maximize copy number. This is highlighted by the discrepancy between the high copy number and relatively low titer of hapten 1, and the fact that the bioconjugates with the two lowest copy numbers, haptens 3 and 4, led to the greatest production of anti-SCRA antibodies while demonstrating the most sensitive detection by competitive ELISA. One possible explanation for this phenomenon could be the occurrence of overlapping haptens with certain bioconjugate conformations at higher copy numbers, causing antibodies to recognize portions of two different haptens as their epitopes. Notably, the relationship between hapten copy number and functional efficacy may also be distinct for differing carrier-proteins [41].

Further elaboration of the relationship between hapten structure and SCRA cross reactivity is also an important priority, as direct structural mimics of specific SCRAs were not always found to be the most effective at producing high-affinity species. The lowest IC_50_ values of both antibody populations were against their cognate SCRA (**3** and **5** for haptens 3 and 4, respectively), with greater IC_50_ increase following 5-fluoronation of the alkyl tails compared to the indole versus indazole core switches. This contrasts with what is observed for hapten 1 in which 5-fluoronation improved SCRA detection, despite the lack data for an indazole version of this hapten. Antibodies against haptens 3 and 4 also displayed binding to **7**, which contains a non-bioisosteric p-fluorobenzyl tail. This result suggests that presentation of the core, linker and head subregions is sufficient to elicit an immune response in mice that creates anti-cognate SCRA antibodies. Furthermore, it is possible that most of the hexanoic tails of the haptens are sterically occluded during immune system presentation following vaccination, making this subregion inconsequential during antibody binding. Future experiments using SCRAs with other structural tail changes (e.g., AMB-CHMICA/AMB-CHMINACA) could be undertaken to test this hypothesis.

Despite breakthroughs in the methods of NPS detection, identification of cryptic or novel structures remains a challenge in regular clinical practice, making this another priority area for future improvements. Many of these methods require knowledge of the NPS/metabolite structure, which is particularly difficult for illicit drug products that often contain a multitude of unknown SCRAs and other contaminants. Therefore, assessment of cross-reactivity limits for a larger pool of hapten/SCRA pairs, especially those that include recent structural variations such as tert-leucine head regions, azaindole cores, or cumyl linkers, will be critical to test the generalizability of the chemoinformatic-informed approach reported here. Such advancements in understanding of cross-reactivity immunopharmacological approaches for detecting and mitigating the effects of novel SCRAs could also translate to more broadly improved understanding of the relationship between haptenic structure and antibody function for additional clinically relevant NPS classes

Finally, although the results with pre-immunization for blocking SCRA effects in vivo are a promising proof of concept, this prophylactic approach is not likely to be a realistic approach for clinical application. Therefore, the generation of optimized monoclonal anti-SCRA antibodies is imperative to maximize both their diagnostic and therapeutic potential.

## 5. Conclusions

As NPS compounds continue to evolve through clandestine medicinal chemistry efforts, the methods of identifying these compounds during outbreaks must evolve as well. This is especially true for SCRAs, which share many bioisosteric similarities among the compounds, making the prioritization of reliable cross-reactivity essential for the utility of any immune-based approach employed for diagnostic or therapeutic purposes. This work describes the synthesis of four haptens, three of which were able to generate high titers of anti-SCRA antibodies with micromolar to sub-micromolar affinity against multiple SCRA compounds with an MCS structural similarity score of 0.6 or above, in comparison to the hapten itself. This resulted in broad detection of multiple structurally related SCRAs at concentrations below 10 ng/mL, including newer generations of SCRAs that evade detection by current commercial ELISA kits. The polyclonal sera developed in this study were also sufficient to demonstrate functional blockade of SCRA-induced toxicologic effects in mice at doses of 1–3 mg/kg, indicating the potential to use antibody-based strategies for symptom mitigation in the context of SCRA intoxication.

Overall, these results indicate that anti-SCRA haptens can yield functionally relevant antibodies against multiple related compounds within and across SCRA generations, and that it may be possible to define general regions of likely antibody cross-reactivity within chemical spaces using readily accessible chemoinformatic information. These findings are especially relevant in the face of increasing numbers of otherwise unknown or cryptic SCRA analogues, as they provide a basis for further development of multiplexed SCRA ELISA screening panels that can provide actionable information about SCRA generational and structural identity within minutes to hours, rather than days to weeks. Based on the ability of the anti-SCRA antibodies reported here to blunt negative effects of SCRAs, further value may be found by combining this multiplexed screening approach with the development of monoclonal antibodies for rapid SCRA overdose reversal to yield an integrated rapid detection and intervention platform to address future SCRA intoxication outbreaks.

## Figures and Tables

**Table 1:** IC_50_ and % inhibition (10 μM) values of each hapten against our SCRA panel.

	HAPTEN 1		HAPTEN 2		HAPTEN 3		HAPTEN 4	
	IC_50_ (μM)	% Inhibition	IC_50_ (μM)	% Inhibition	IC_50_ (μM)	% Inhibition	IC_50_ (μM)	% Inhibition
**1**	5.541	60.0	-	17.7	-	14.6	-	10.9
**2**	1.136	79.3	-	29.5	-	18.0	-	14.4
**3**	-	0.0	-	15.2	0.038	91.8	1.256	71.0
**4**	-	8.8	-	19.9	0.744	69.0	6.690	54.3
**5**	-	6.9	-	25.5	0.269	82.6	0.334	85.5
**6**	-	16.8	-	28.7	3.324	57.4	2.656	63.9
**7**	-	11.1	-	30.7	0.299	76.0	0.966	77.7
**8**	-	13.6	-	31.5	-	19.2	-	11.0

## Data Availability

Data available in a publicly accessible repository. The data presented in this study are openly available in FigShare at 10.6084/m9.figshare.20425410.

## Supplementary Materials

The following supporting information can be downloaded at: https://www.mdpi.com/article/10.3390/vaccines10081253/s1, Chemistry: General Syntheses; Hapten Syntheses; SCRA Syntheses; Figure S1: MALDI-ToF analysis of BSA-hapten1 bioconjugate; Figure S2: MALDI-ToF analysis of BSA-hapten2 bioconjugate; Figure S3: MALDI-ToF analysis of BSA-hapten3 bioconjugate; Figure S4: MALDI-ToF analysis of BSA-hapten4 bioconjugate; Figure S5: Midpoint antibody titers for pooled sera collected on day 21 from all four vaccine groups; Figure S6: Percent inhibitions of our SCRA panel against day 35 pooled sera; Figure S7: Percent inhibition versus AP (left) and MCS (right) Tanimoto coefficients of structural similarity. Analysis was performed for each hapten-SCRA pair in this research (32) and for each pair that demonstrated measurable cross-reactivity from the Lin et. al. data set; Figure S8: Hot plate and catalepsy test MPE values for vaccine groups 1–4 (a-d, respectively); Table S1: Atom pair (AP) and maximum common substructure (MCS) Tanimoto coefficients for every hapten-SCRA pair from this work. Structural similarity of the molecules increases as the coefficients approach 1.
