# Supplementary material for: Development of Cross-Reactive Antibodies for the Identification and Treatment of Synthetic Cannabinoid Receptor Agonist Toxicity
Source: Vaccines (Basel). 2022 Aug 4;10(8):1253. doi: 10.3390/vaccines10081253 (PMC9415894; doi:10.3390/vaccines10081253)
Supplement: Supplementary file 1 [file vaccines-10-01253-s001.zip › vaccines-1813521-supplementary.pdf]

Supplementary Information

Development of Cross-Reactive Antibodies for the Identification and Treatment of Synthetic Cannabinoid Receptor Agonist Toxicity

Adam L. Worob and Cody J. Wenthur

Pharmaceutical Sciences Division, University of Wisconsin-Madison School of Pharmacy, Madison, WI 53705, USA

Chemistry: General Syntheses

GS1. $AlCl_3$ Friedel-Crafts acylation: Indole (1 eq) was dissolved in dry DCM and added to a solution of $AlCl_3$ (1 eq) stirring in dry DCM under nitrogen at room temperature. After 45 minutes, naphthoyl chloride (1.33 eq) was added and the reaction continued until indole consumption was observed via TLC (2 hours). The reaction was then diluted with DCM and washed with water and brine and dried over Na_2SO_4. Column chromatography (10% ethyl acetate in hexanes) afforded the indole naphthyl ketone.

GS2. EDC/DMAP coupling reactions: To an N-substituted or unsubstituted indole/indazole-3-carboxylic acid (1eq) solution in a 3:1 DCM:THF solvent system was added DMAP (1 eq), EDC-HCl (1.25 eq) and amine or alcohol head group (1eq) and reacted at room temperature for 18 hours. The solvent was removed via rotary evaporation and the crude material was redissolved in ethyl acetate prior to washing with saturated sodium bicarbonate, water and brine and drying over Na_2SO_4. All coupling reactions were purified via column chromatography to afford the amide or ester products.

GS3. N-alkylation (NaH or sodium tert-butoxide): To a stirring solution of either sodium hydride (3 eq), or sodium tert-butoxide (3 eq) if the starting material contained a methyl ester, in dry DMF under nitrogen, was added indole or indazole starting material (1 eq) dissolved in dry DMF. The reaction proceeded at room temperature for 45 minutes prior to the addition of brominated alkyl or methyl/benzyl ester tail (1.25 eq). The reaction was quenched after 90 minutes with water and extracted three times into ethyl acetate. The organic layers were combined and washed with saturated sodium carbonate solution, water, brine and dried over Na_2SO_4. If full consumption of indole/indazole starting material was indicated by TLC, the alkylated product was used directly in the deprotection step without column chromatography purification.

GS4. Pd/C mediated benzyl ester hydrogenolysis: To a stirring solution of benzyl ester containing intermediate (1 eq) in ethanol was added 10% Pd/C. Nitrogen was then bubbled through the solution for five minutes while the hydrogen balloon was obtained. Hydrogen was then bubbled through the reaction for one hour before diluting the reaction with ethanol. Filtration through celite and rotary evaporation afforded crude acid which was subsequently purified via column chromatography.

GS5. Carboxylic acid methyl ester protection: To a solution of indole or indazole-3-carboxylic acid (1 eq) in methanol (.2M final concentration) was added tosylic acid (.5 eq). The reaction proceeded at reflux until acid consumption was observed via TLC. The solution was then neutralized, and the solvent was removed via rotary evaporation before resuspending the crude ester in ethyl acetate. The organic solution was washed with water and brine before being dried over Na_2SO_4. Column chromatography was performed to purify the ester intermediates.

GS6. Methyl ester hydrolysis: Ester-containing intermediates were dissolved in THF prior to the addition of 2M potassium hydroxide solution (8 eq). Methanol was added until miscibility was observed, and the reaction proceeded overnight at room temperature. The reaction was then quenched with saturated ammonium chloride solution and extracted three times with ethyl acetate. The organic layers were combined and washed with water and brine and dried over Na_2SO_4. The crude material was used directly in the next coupling reaction except in the case of hapten 1 in which ester hydrolysis was the final step, requiring column chromatography for purification.

Hapten Syntheses:

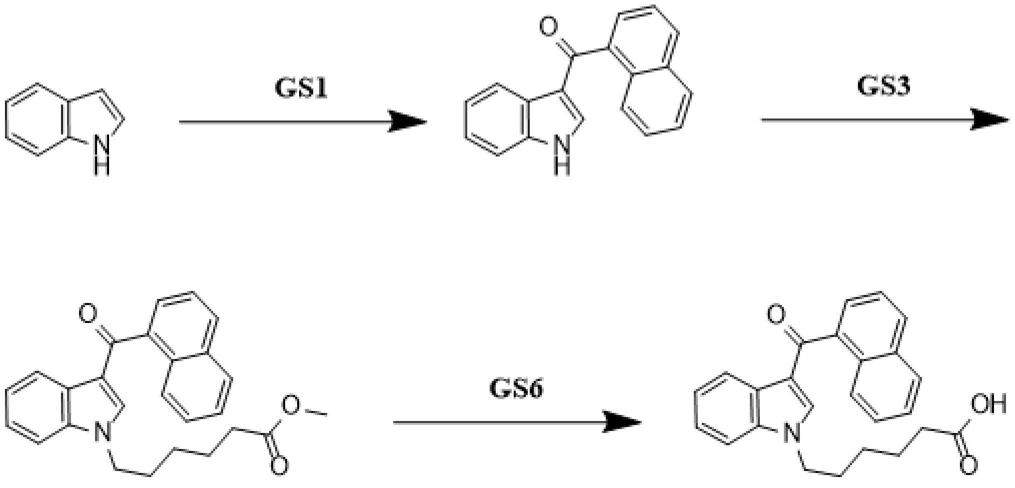

Hapten 1: ^{1}H NMR (400 MHz, DMSO) δ 8.36 – 8.26 (m, 1H), 8.09 (dd, *J* = 8.1, 1.2 Hz, 1H), 8.02 (td, *J* = 8.5, 1.4 Hz, 2H), 7.78 (s, 1H), 7.69 (dd, *J* = 7.1, 1.4 Hz, 1H), 7.69 – 7.59 (m, 2H), 7.55 (dddd, *J* = 21.5, 8.3, 6.8, 1.5 Hz, 2H), 7.33 (dtd, *J* = 12.7, 7.1, 1.5 Hz, 2H), 4.20 (t, *J* = 7.2 Hz, 2H), 2.06 (t, *J* = 7.3 Hz, 2H), 1.71 (p, *J* = 7.4 Hz, 2H), 1.45 (p, *J* = 7.4 Hz, 2H), 1.21 (tt, *J* = 9.5, 6.1 Hz, 2H).^{13}C NMR (101 MHz, DMSO) δ 191.53, 174.99, 139.81, 138.88, 137.25, 133.77, 130.49, 130.23, 128.78, 127.23, 126.81, 126.79, 126.26, 125.67, 125.48, 123.89, 123.03, 122.17, 116.51, 111.48, 46.53, 33.94, 29.55, 26.03, 24.38. HRMS m/z [M+H^{+}] calculated = 386.1751, found = 386.1751. .

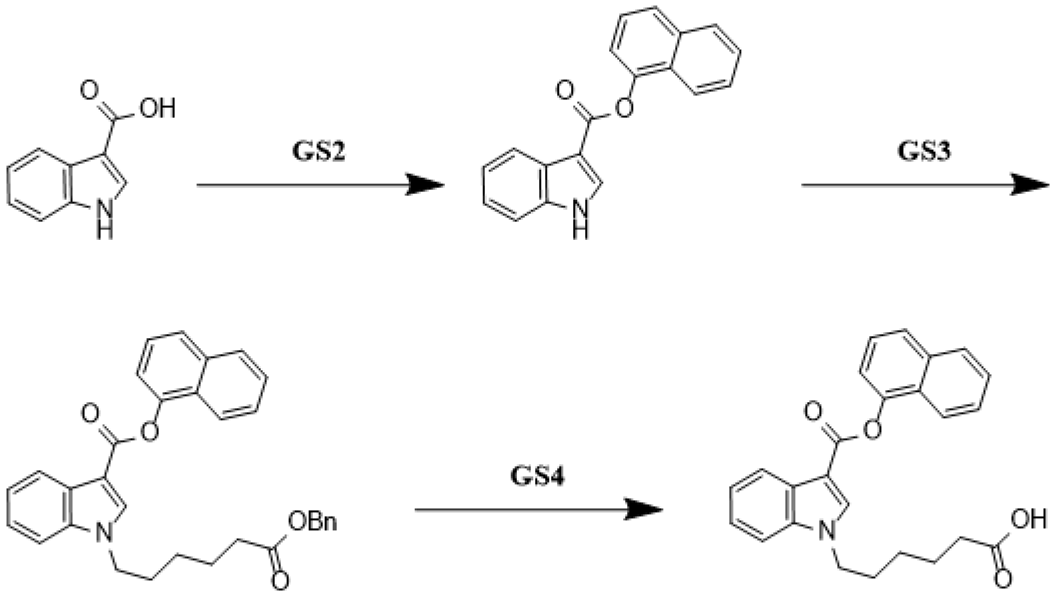

Hapten 2: ^{1}H NMR (400 MHz, DMSO) δ 8.60 (s, 1H), 8.12 – 8.04 (m, 1H), 8.03 (dt, *J* = 7.7, 2.4 Hz, 1H), 7.97 – 7.86 (m, 2H), 7.71 (d, *J* = 8.1 Hz, 1H), 7.67 – 7.56 (m, 2H), 7.60 – 7.52 (m, 1H), 7.51 – 7.40 (m, 1H), 7.38 – 7.25 (m, 2H), 4.36 (t, *J* = 7.1 Hz, 2H), 2.27 – 2.10 (m, 2H), 1.88 (p, *J* = 8.0 Hz, 2H), 1.56 (dp, *J* = 14.4, 7.5 Hz, 2H), 1.42 – 1.27 (m, 2H).^{13}C NMR (101 MHz, DMSO) δ 174.90, 162.98, 146.93, 137.37, 137.02, 134.69, 128.48, 127.52, 127.12, 127.02, 126.90, 126.29, 126.04, 123.31, 122.55, 121.67, 121.12, 119.42, 111.76, 104.47, 46.70, 34.04, 29.77, 26.22, 24.55. HRMS m/z [M+H^{+}] calculated = 402.1700, found = 402.1680. .

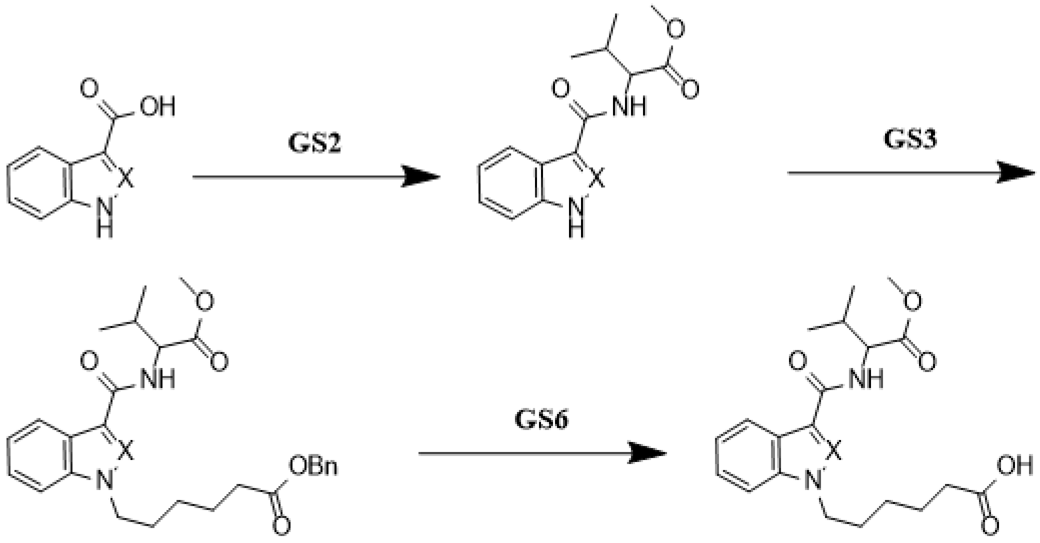

Hapten 3 (X=CH): ^{1}H NMR (400 MHz, DMSO) δ 12.04 (s, 1H), 8.29 (s, 1H), 8.12 (dd, *J* = 7.8, 1.2 Hz, 1H), 7.93 (d, *J* = 8.0 Hz, 1H), 7.54 (d, *J* = 8.1 Hz, 1H), 7.20 (ddd, *J* = 8.3, 7.0, 1.3 Hz, 1H), 7.13 (td, *J* = 7.5, 1.1 Hz, 1H), 4.36 (t, *J* = 7.6 Hz, 1H), 4.26 – 4.14 (m, 2H), 3.67 (s, 3H), 2.21 (t, *J* = 7.3 Hz, 2H), 2.15 (dt, *J* = 13.6, 6.8 Hz, 1H), 1.82 (p, *J* = 7.2 Hz, 2H), 1.56 (p, *J* = 7.5 Hz, 2H), 1.31 (tdd, *J* = 11.2, 9.2, 4.9 Hz, 2H), 1.03 – 0.92 (m, 6H). ^{13}C NMR (101 MHz, DMSO) δ 174.97, 173.31, 165.10, 136.50, 131.82, 127.09, 122.53, 121.66, 121.21, 110.78, 109.15, 58.17, 52.06, 46.28, 35.50, 33.96, 32.36, 30.03, 27.53, 26.22, 19.54. HRMS m/z [M+H^{+}] calculated = 389.2071, found = 389.2074. .

Hapten 4 (X=N): ^{1}H NMR (400 MHz, DMSO) δ 8.14 (d, *J* = 1.1 Hz, 1H), 8.08 (d, *J* = 8.3 Hz, 1H), 7.82 – 7.74 (m, 1H), 7.47 (ddd, *J* = 8.4, 6.9, 1.1 Hz, 1H), 7.28 (ddd, *J* = 7.9, 6.8, 0.8 Hz, 1H), 4.51 (t, *J* = 7.1 Hz, 2H), 4.44 (dd, *J* = 8.3, 6.7 Hz, 1H), 3.70 (s, 3H), 2.26 (q, *J* = 6.8 Hz, 1H), 2.19 (t, *J* = 7.3 Hz, 2H), 1.89 (p, *J* = 7.3 Hz, 2H), 1.55 (p, *J* = 7.3 Hz, 2H), 1.35 – 1.25 (m, 2H), 0.96 (t, *J* = 6.8 Hz, 6H). ^{13}C NMR (101 MHz, DMSO) δ 174.82, 172.48, 162.41, 141.00, 136.65, 127.13, 123.01, 122.56, 122.06, 110.94, 57.73, 52.33, 49.10, 34.00, 30.35, 29.63, 26.19, 24.51, 19.56, 19.14. HRMS m/z [M+H^{+}] calculated = 390.2018, found = 390.2020. .

SCRA Syntheses:

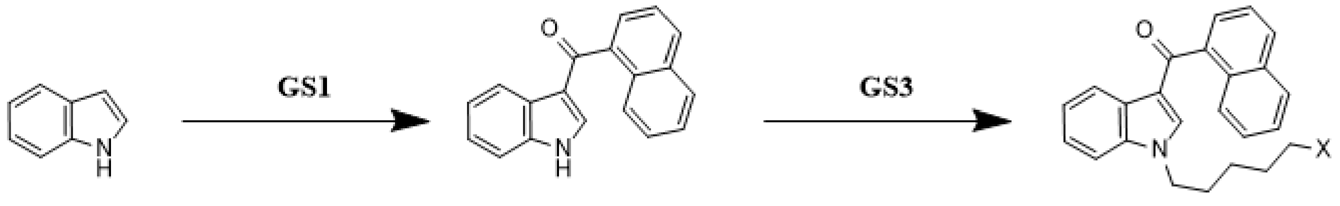

Scheme 1. – JWH-018 (X=H): ^{1}H NMR (400 MHz, DMSO) δ 8.35 – 8.26 (m, 1H), 8.09 (d, *J* = 8.1 Hz, 1H), 8.02 (t, *J* = 7.8 Hz, 2H), 7.78 (s, 1H), 7.69 (dd, *J* = 7.0, 1.4 Hz, 1H), 7.67 – 7.59 (m, 2H), 7.55 (dddd, *J* = 21.4, 8.3, 6.8, 1.5 Hz, 2H), 7.32 (tt, *J* = 7.2, 5.6 Hz, 2H), 4.19 (t, *J* = 7.2 Hz, 2H), 2.05 (t, *J* = 7.3 Hz, 2H), 1.71 (p, *J* = 7.5 Hz, 2H), 1.44 (p, *J* = 7.4 Hz, 2H), 1.26 – 1.14 (m, 2H). ^{13}C NMR (101 MHz, $CDCl_3$) δ 192.08, 139.13, 137.99, 137.06, 133.76, 130.82, 129.98, 128.18, 127.01, 126.77, 126.31, 126.02, 125.85, 124.58, 123.62, 122.94, 122.88, 117.56, 110.01, 47.19, 29.50, 28.92, 22.18, 13.88. HRMS m/z $[M+H^+]$ calculated = 342.1852, found = 342.1860. .

Scheme 2. – AM-2201 (X=F): ^{1}H NMR (400 MHz, $CDCl_3$) δ 8.53 – 8.44 (m, 1H), 8.22 – 8.15 (m, 1H), 7.97 (dt, *J* = 8.2, 1.1 Hz, 1H), 7.94 – 7.88 (m, 1H), 7.66 (dd, *J* = 7.0, 1.3 Hz, 1H), 7.57 – 7.47 (m, 2H), 7.47 (ddd, *J* = 8.2, 6.8, 1.5 Hz, 1H), 7.43 – 7.32 (m, 4H), 4.44 (t, *J* = 5.9 Hz, 1H), 4.32 (t, *J* = 5.9 Hz, 1H), 4.11 (dt, *J* = 13.6, 7.2 Hz, 2H), 1.86 (p, *J* = 7.3 Hz, 2H), 1.76 – 1.58 (m, 2H), 1.41 (tt, *J* = 9.7, 6.3 Hz, 2H). ^{13}C NMR (101 MHz, $CDCl_3$) δ 192.04, 139.07, 137.83, 136.99, 133.77, 130.81, 130.02, 128.20, 127.03, 126.78, 126.32, 125.99, 125.87, 124.58, 123.71, 123.02, 122.94, 117.71, 109.91, 82.75, 47.03, 29.96, 29.48, 22.79. HRMS m/z $[M+H^+]$ calculated = 360.1758, found = 342.1762. .

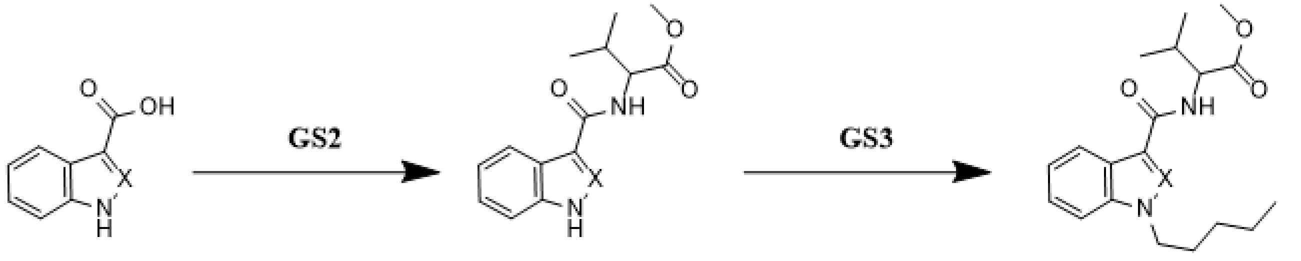

Scheme 3. – MMB-PICA (X=CH): ^{1}H NMR (400 MHz, $CDCl_3$) δ 7.99 (dt, *J* = 7.8, 2.9 Hz, 1H), 7.75 (s, 1H), 7.44 – 7.35 (m, 1H), 7.33 – 7.25 (m, 2H), 6.47 (d, *J* = 8.7 Hz, 1H), 4.87 (dd, *J* = 8.7, 4.8 Hz, 1H), 4.14 (t, *J* = 7.2 Hz, 2H), 3.79 (s, 3H), 2.39 – 2.23 (m, 1H), 1.87 (p, *J* = 7.2 Hz, 2H), 1.33 (qd, *J* = 8.3, 4.7 Hz, 4H), 1.04 (dd, *J* = 9.5, 6.9 Hz, 6H), 0.89 (t, *J* = 6.8 Hz, 3H). ^{13}C NMR (101 MHz, $CDCl_3$) δ 172.14, 163.90, 135.60, 130.82, 124.32, 121.39, 120.53, 119.04, 109.45, 109.33, 55.87, 51.14, 45.87, 30.70, 28.64, 27.98, 21.25, 18.08, 17.07, 12.87. HRMS m/z $[M+H^+]$ calculated = 345.2173, found = 345.2178. .

Scheme 5. – MMB-PINACA (X=N): ^{1}H NMR (400 MHz, $CDCl_3$) δ 8.35 (dt, *J* = 8.2, 1.1 Hz, 1H), 7.84 (ddt, *J* = 22.1, 8.6, 1.1 Hz, 1H), 7.51 – 7.34 (m, 2H), 7.38 – 7.20 (m, 1H), 4.91 – 4.74 (m, 2H), 4.39 (t, *J* = 7.3 Hz, 1H), 3.83 (s, 1H), 3.78 (s, 2H), 2.43 – 2.23 (m, 1H), 1.97 (hept, *J* = 6.7 Hz, 2H), 1.46 – 1.29 (m, 2H), 1.26 (s, 2H), 1.11 – 1.00 (m, 6H), 0.87 (s, 3H). ^{13}C NMR (101 MHz, $CDCl_3$) δ 172.43, 162.61, 140.82, 136.61, 126.60, 122.91, 122.78, 122.54, 109.25, 56.76, 52.14, 49.48, 31.67, 29.19, 28.15, 22.25, 19.16, 18.04, 13.93. HRMS m/z $[M+H^+]$ calculated = 346.2125, found = 346.2127.

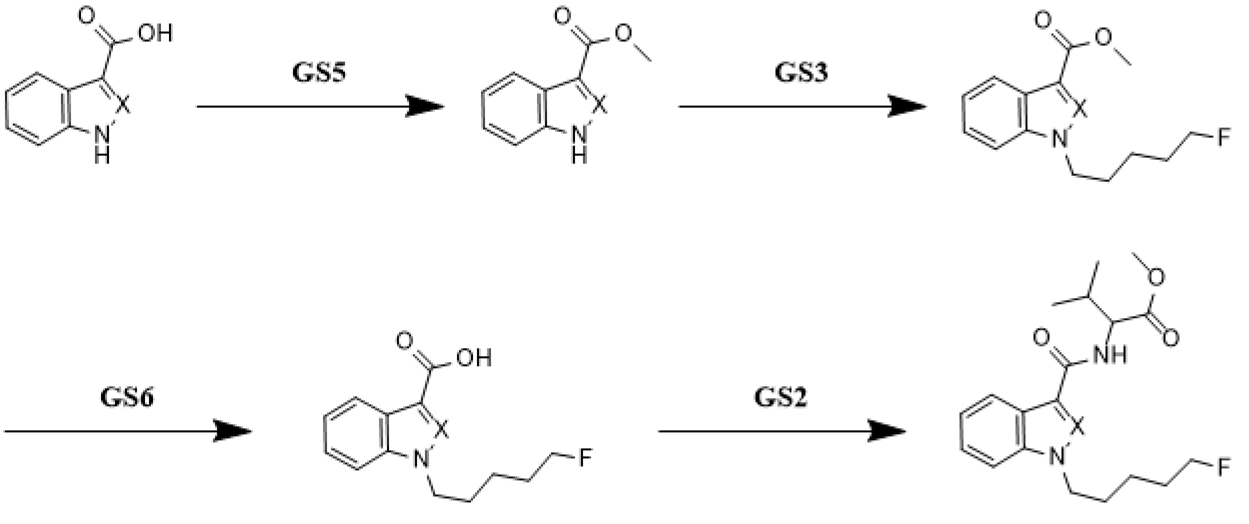

Scheme 4. – 5F-MMB-PICA (X=CH): ^{1}H NMR (400 MHz, $CDCl_3$) δ 8.00 (dt, *J* = 7.9, 2.9 Hz, 1H), 7.74 (s, 1H), 7.43 – 7.35 (m, 1H), 7.34 – 7.26 (m, 2H), 6.47 (d, *J* = 8.7 Hz, 1H), 4.87 (dd, *J* = 8.7, 4.8 Hz, 1H), 4.48 (t, *J* = 5.9 Hz, 1H), 4.37 (t, *J* = 5.9 Hz, 1H), 4.17 (t, *J* = 7.1 Hz, 2H), 3.79 (s, 3H), 2.37 – 2.23 (m, 1H), 1.93 (p, *J* = 7.3 Hz, 2H), 1.81 – 1.63 (m, 2H), 1.47 (tt, *J* = 10.5, 6.3 Hz, 2H), 1.04 (dd, *J* = 9.8, 6.9 Hz, 6H). ^{13}C NMR (101 MHz, $CDCl_3$) δ 173.18, 164.92, 136.59, 131.76, 125.38, 122.56, 121.67, 120.15, 110.65, 110.28, 84.48, 56.93, 52.20, 46.73, 31.71, 30.05, 29.65, 22.84, 19.12, 18.10. HRMS m/z [M+H$^+$] calculated = 363.2078, found = 363.2082.

Scheme 6. – 5F-AMB (X=N): ^{1}H NMR (400 MHz, $CDCl_3$) δ 8.35 (dt, *J* = 8.2, 1.0 Hz, 1H), 7.50 – 7.40 (m, 2H), 7.44 – 7.32 (m, 1H), 7.32 – 7.21 (m, 1H), 4.81 (dd, *J* = 9.1, 5.2 Hz, 1H), 4.50 (t, *J* = 6.0 Hz, 1H), 4.42 (t, *J* = 7.1 Hz, 2H), 4.38 (t, *J* = 6.0 Hz, 1H), 3.78 (s, 3H), 2.44 – 2.23 (m, 1H), 2.02 (p, *J* = 7.3 Hz, 2H), 1.84 – 1.60 (m, 2H), 1.54 – 1.39 (m, 2H), 1.05 (dd, *J* = 6.8, 6.1 Hz, 6H). ^{13}C NMR (101 MHz, $CDCl_3$) δ 172.65, 162.54, 140.83, 136.76, 126.76, 122.92, 122.84, 122.67, 109.14, 83.73, 56.76, 52.16, 49.20, 31.60, 29.84, 29.33, 22.69, 19.15, 18.06. HRMS m/z [M+H$^+$] calculated = 364.2031, found = 363.2082.

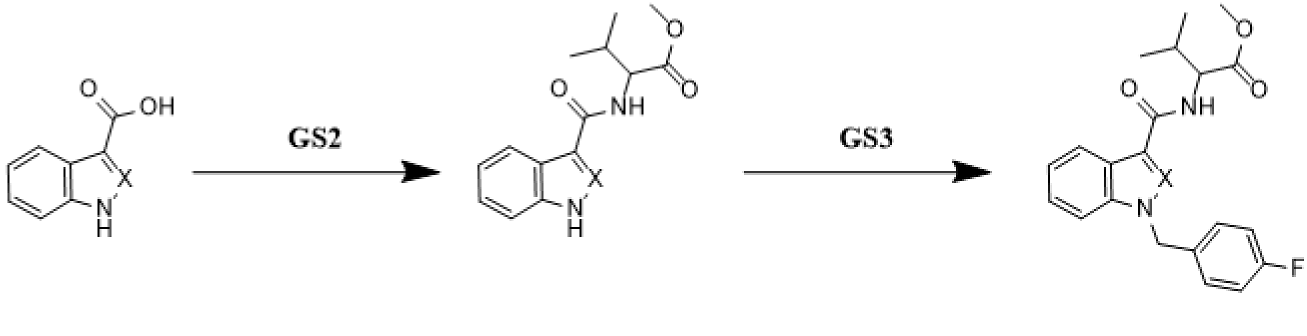

Scheme 7. – AMB-FUBINACA: ^{1}H NMR (400 MHz, $CDCl_3$) δ 8.39 (dt, *J* = 8.2, 1.1 Hz, 1H), 7.51 (d, *J* = 9.0 Hz, 1H), 7.46 – 7.35 (m, 1H), 7.39 – 7.26 (m, 2H), 7.26 – 7.17 (m, 2H), 7.10 – 6.96 (m, 2H), 5.62 (s, 2H), 4.83 (dd, *J* = 9.1, 5.2 Hz, 1H), 3.81 (s, 3H), 2.34 (pd, *J* = 6.9, 5.2 Hz, 1H), 1.07 (t, *J* = 6.7 Hz, 6H). ^{13}C NMR (101 MHz, $CDCl_3$) δ 172.61, 162.39, 161.25, 140.79, 137.33, 131.73, 129.01, 128.92, 127.10, 123.32, 122.94, 122.90, 115.92, 115.71, 109.45, 56.81, 52.93, 52.19, 31.61, 19.16, 18.10. HRMS m/z [M+H$^+$] calculated = 384.1718, found = 384.1712.

Scheme 8. ^{1}H NMR (400 MHz, $CDCl_3$) δ 8.43 – 8.28 (m, 1H), 8.15 (s, 1H), 8.06 (ddd, *J* = 7.7, 1.9, 0.8 Hz, 1H), 7.96 – 7.89 (m, 1H), 7.80 (dt, *J* = 8.4, 1.1 Hz, 1H), 7.60 – 7.28 (m, 7H), 4.26 (t, *J* = 7.2 Hz, 2H), 2.05 – 1.86 (m, 2H), 1.42 (dq, *J* = 8.9, 3.2 Hz, 4H), 0.98 – 0.94 (m, 3H). ^{13}C NMR (101 MHz, $CDCl_3$) δ 163.35, 146.91, 136.79, 135.42, 134.76, 128.00, 127.59, 127.00, 126.34, 125.69, 125.57, 124.96 123.05, 122.32, 122.00, 121.68, 118.65, 110.25, 105.96, 47.30, 29.65, 29.05, 22.30, 13.95. HRMS m/z [M+H$^+$] calculated = 358.1802, found =358.1803.

Supplementary Figures:

Supplementary Figure S1. MALDI-ToF analysis of BSA-hapten1 bioconjugate.

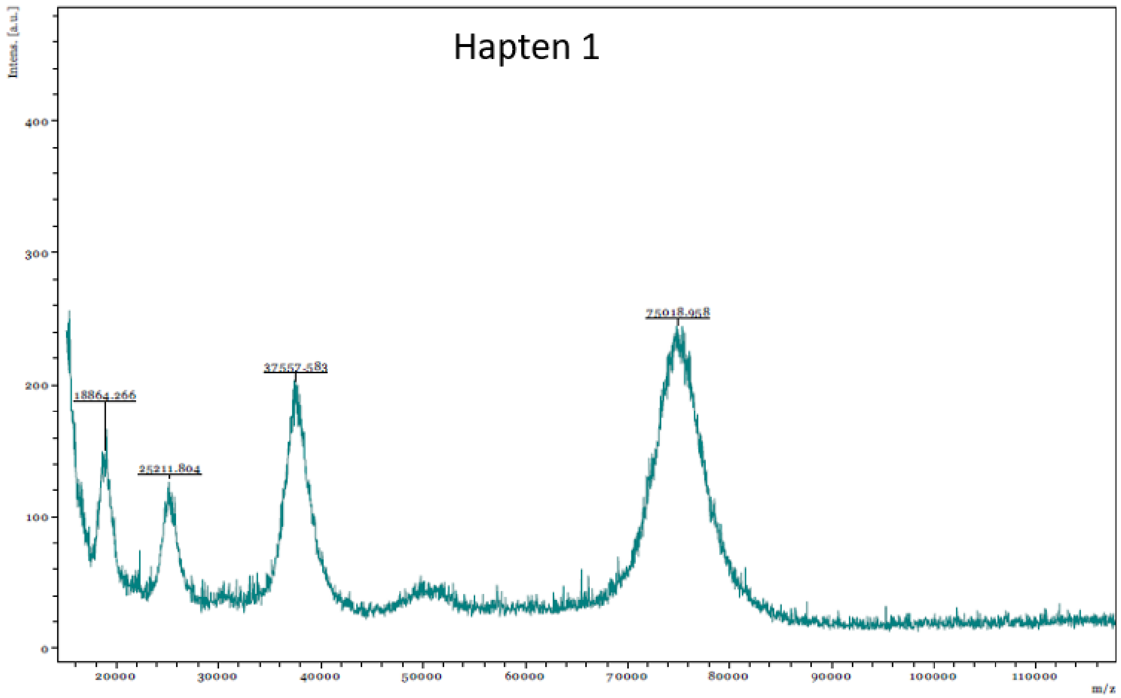

Supplementary Figure S2. MALDI-ToF analysis of BSA-hapten2 bioconjugate.

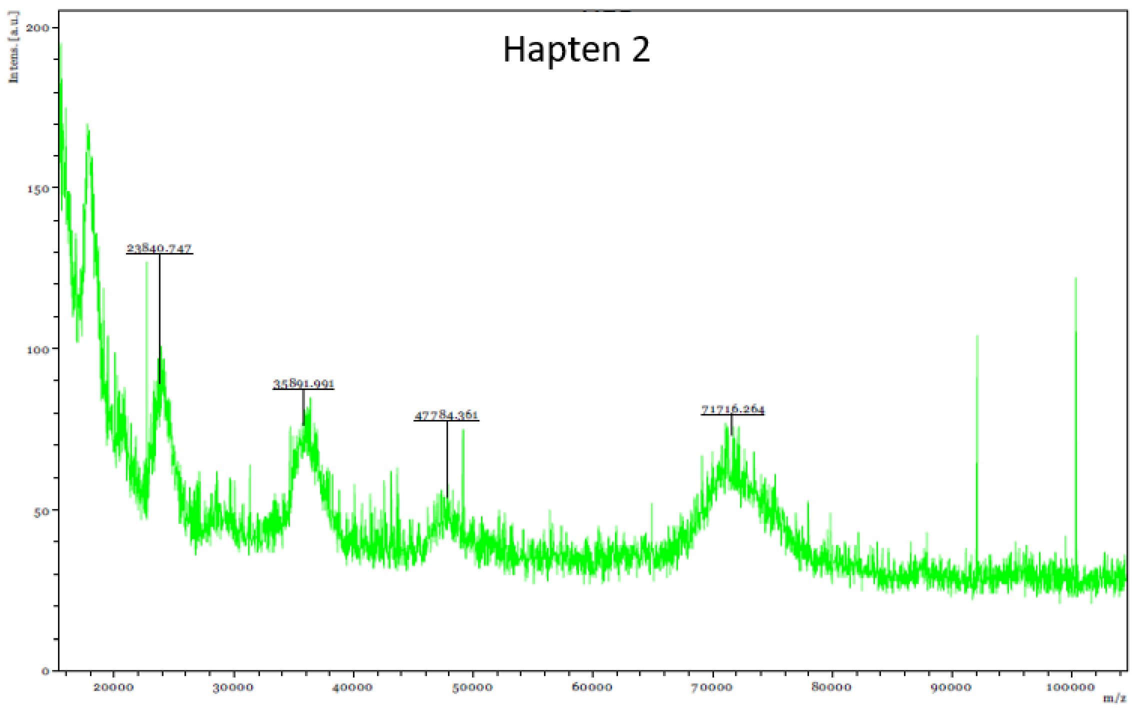

Supplementary Figure S3. MALDI-ToF analysis of BSA-hapten3.

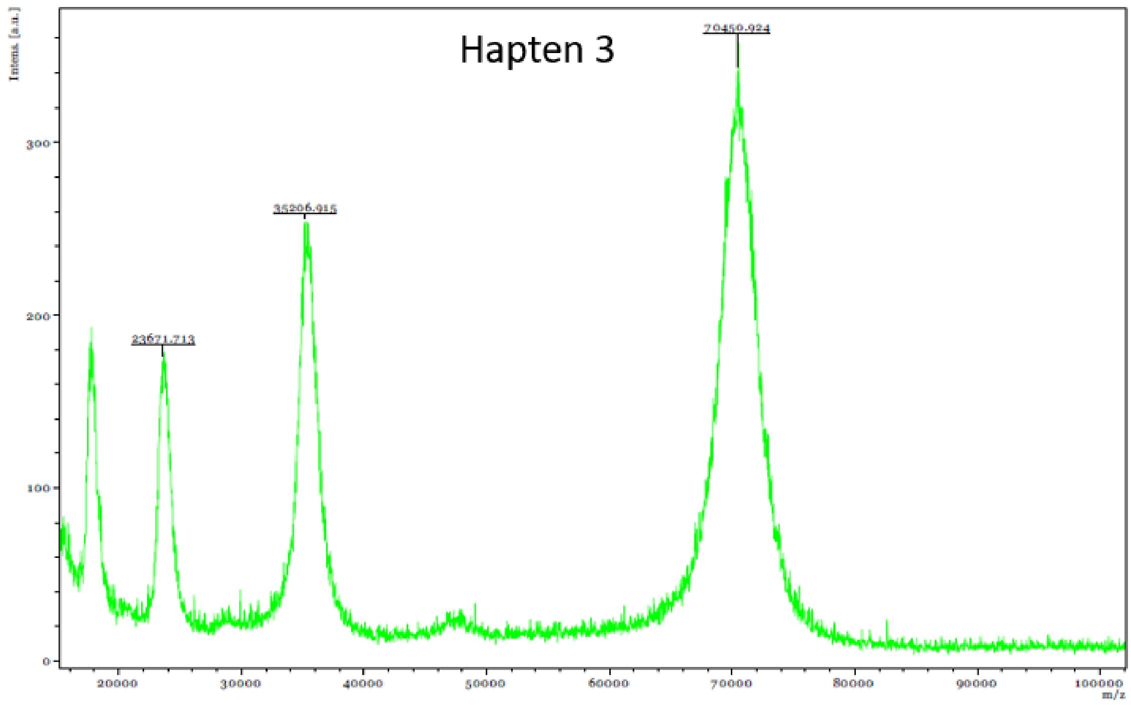

Supplementary Figure S4. MALDI-ToF analysis of BSA-hapten4.

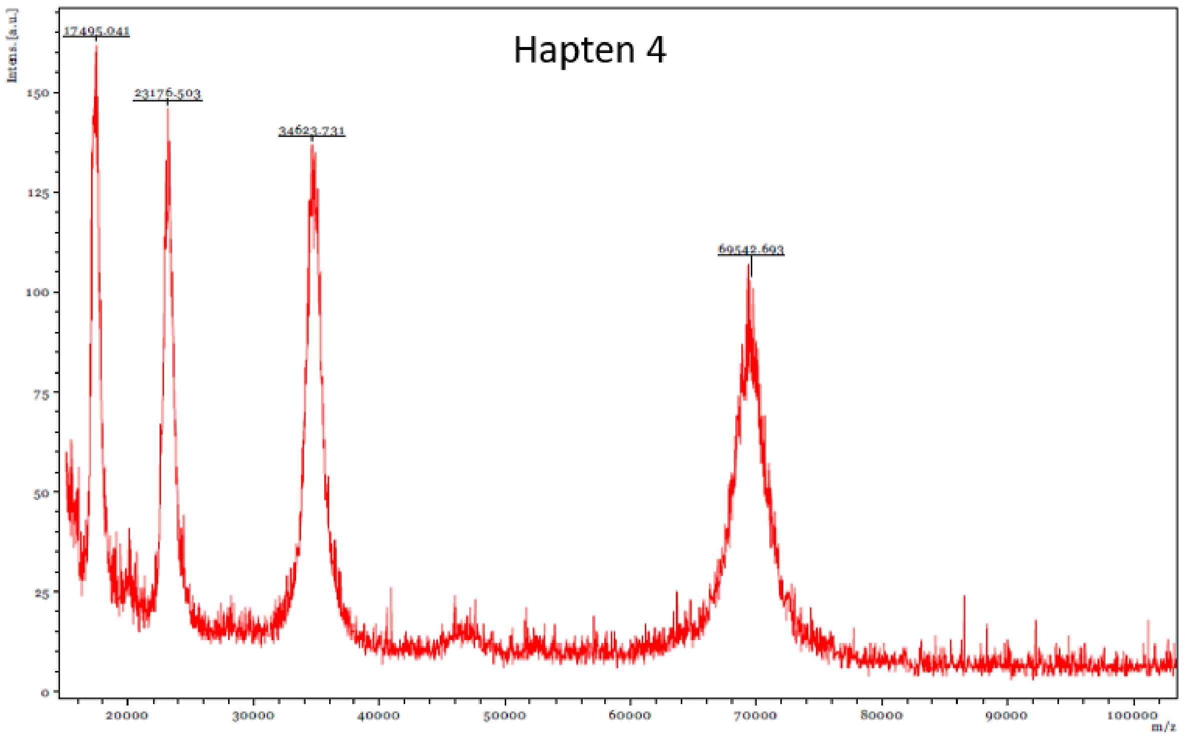

Supplementary Figure S5. Midpoint antibody titers for pooled sera collected on day 21 from all four vaccine groups.

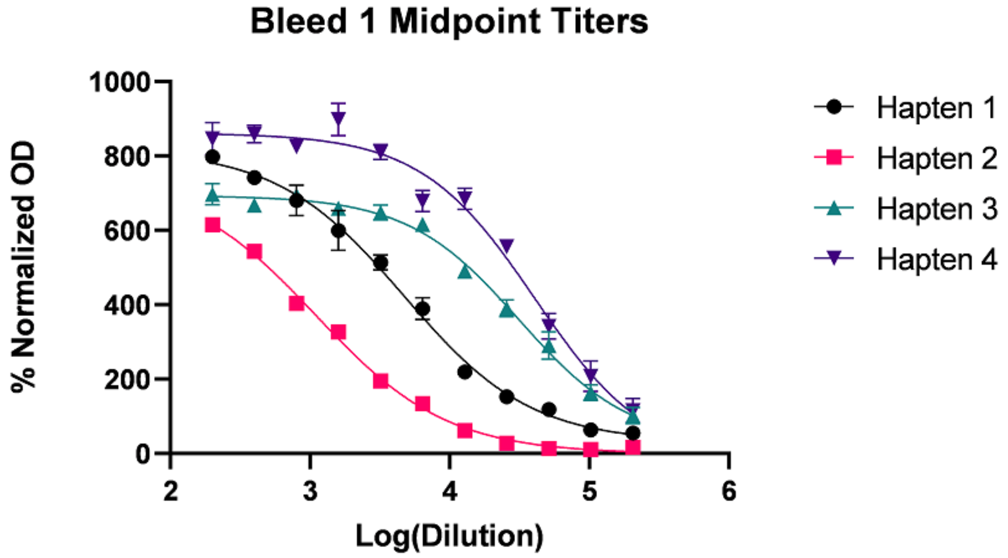

Supplementary Figure S6. Percent inhibitions of our SCRA panel against day 35 pooled sera.

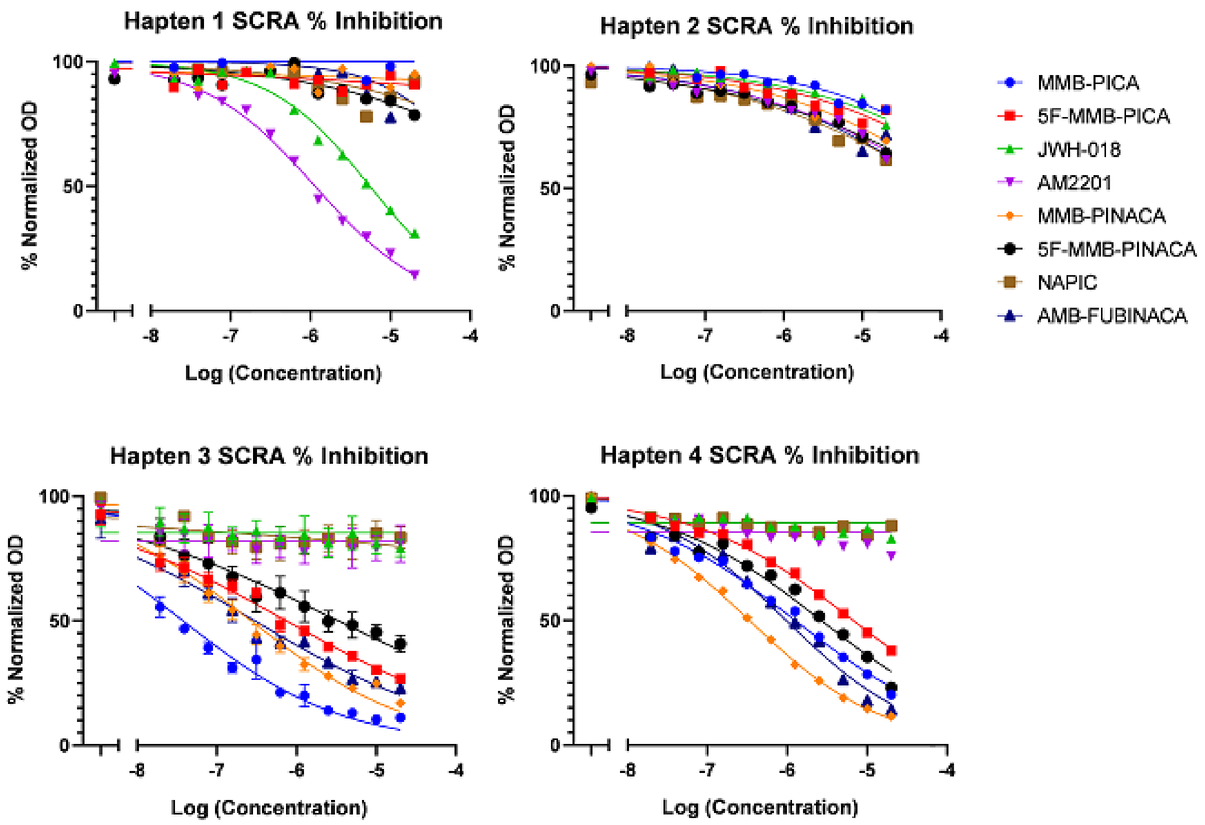

Supplementary Table S1. Atom pair (AP) and maximum common substructure (MCS) Tanimoto coefficients for every hapten-SCRA pair from this work. Structural similarity of the molecules increases as the coefficients approach 1.

	Hapten 1		Hapten 2		Hapten 3		Hapten 4	
	AP	MCS	AP	MCS	AP	MCS	AP	MCS
**1**	0.696	**0.897**	0.542	0.400	0.255	0.421	0.214	0.385
**2**	0.752	**0.867**	0.569	0.390	0.238	0.410	0.201	0.375
**3**	0.217	0.421	0.223	0.410	0.687	**0.893**	0.595	**0.828**
**4**	0.239	0.410	0.244	0.400	0.744	**0.862**	0.646	**0.800**
**5**	0.185	0.385	0.189	0.375	0.599	**0.828**	0.687	**0.893**
**6**	0.204	0.375	0.208	0.366	0.646	**0.800**	0.744	**0.862**
**7**	0.231	0.267	0.230	0.261	0.403	**0.600**	0.426	**0.647**
**8**	0.567	0.400	0.705	**0.900**	0.259	0.410	0.219	0.375

Supplementary Figure S7. Percent inhibition versus AP (left) and MCS (right) Tanimoto coefficients of structural similarity. Analysis was performed for each hapten-SCRA pair in this research (32) and for each pair that demonstrated measurable cross-reactivity from the Lin et. al. data set.

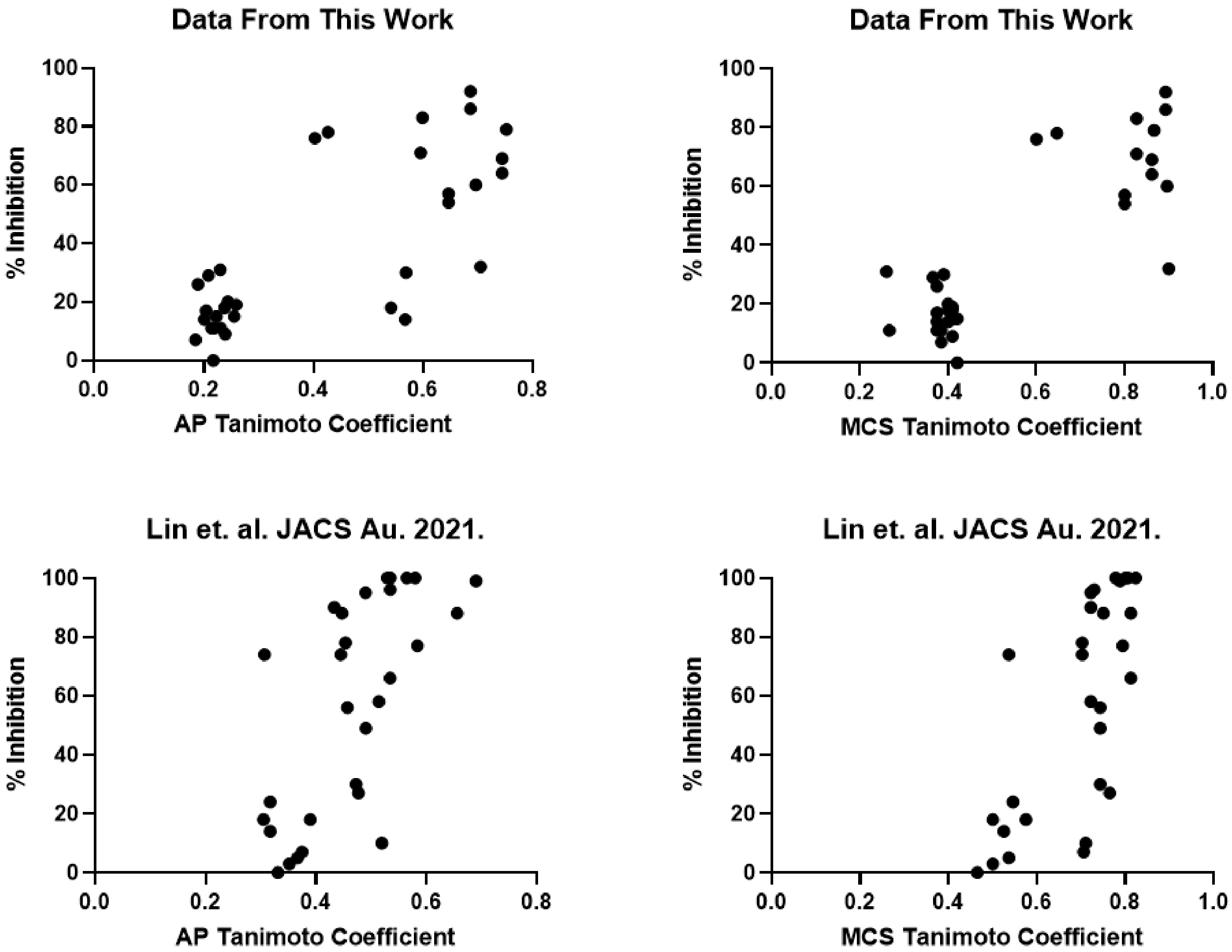

Supplementary Figure S8. Hot plate and catalepsy test MPE values for vaccine groups 1-4 (a-d, respectively).

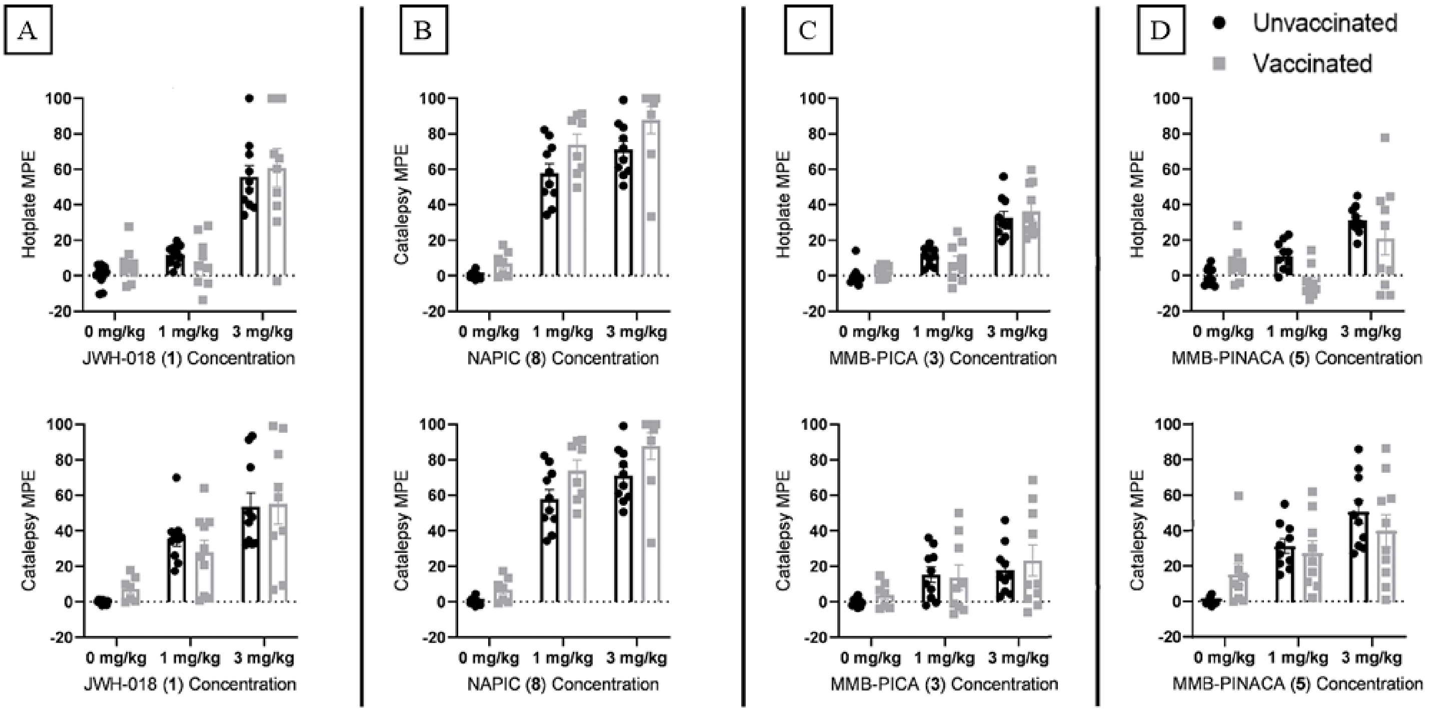

NMR Spectra – Haptens:

Hapten 1

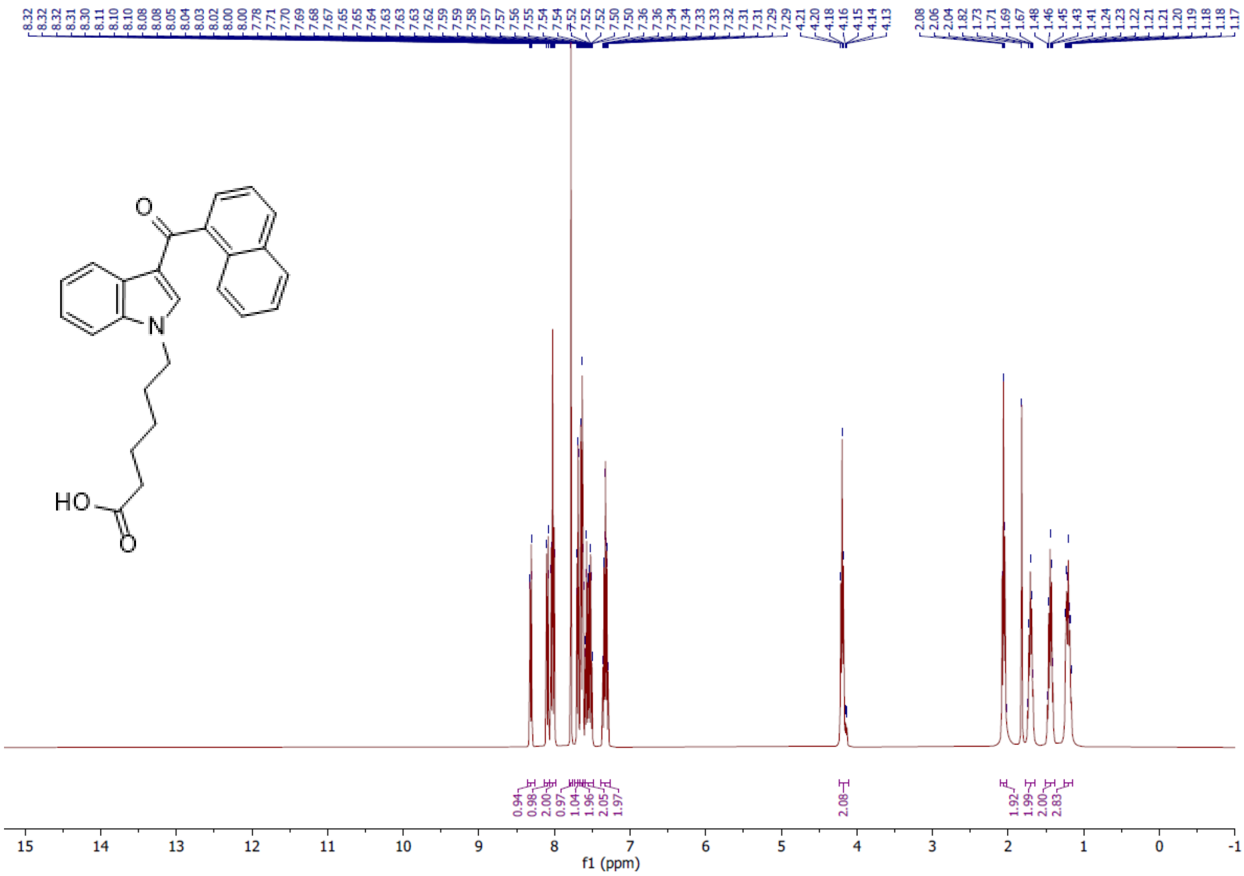

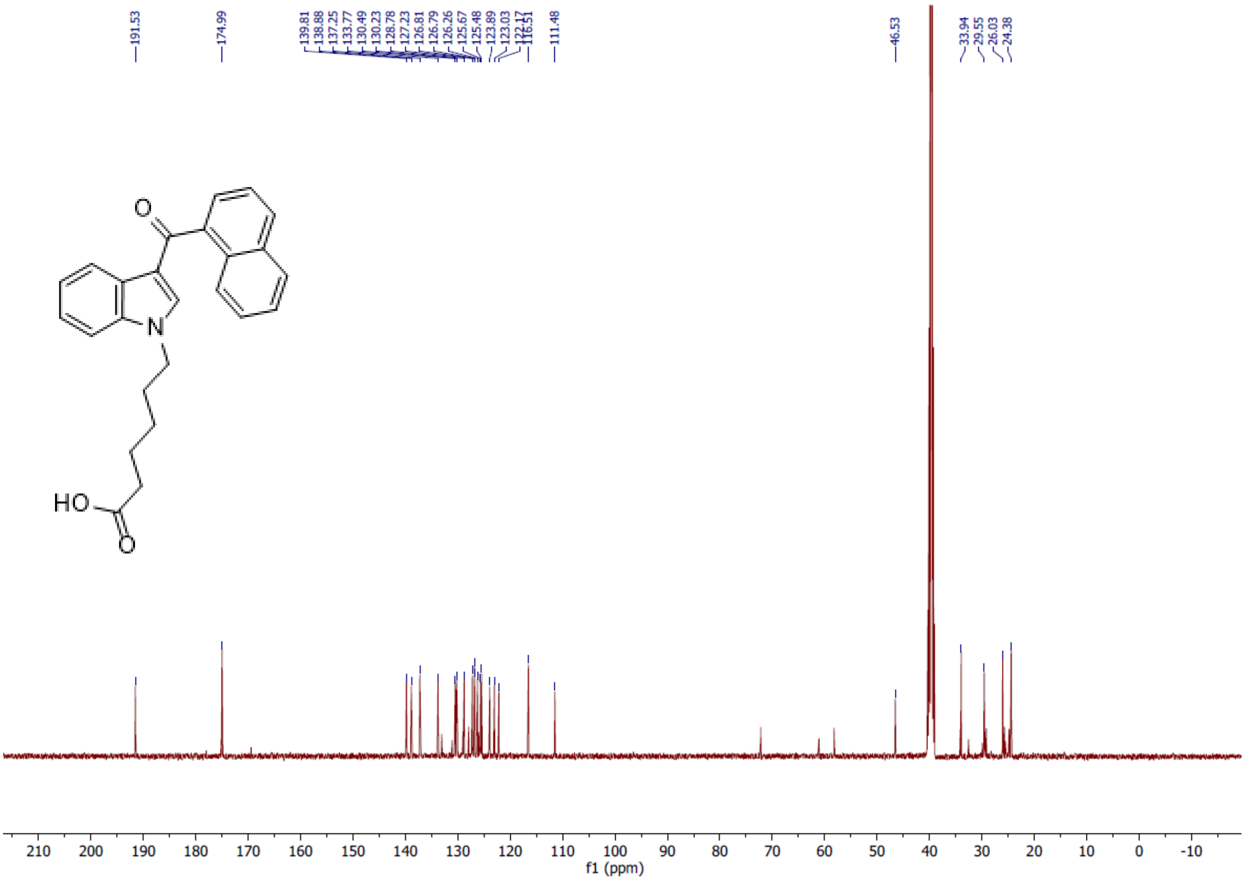

Hapten 2

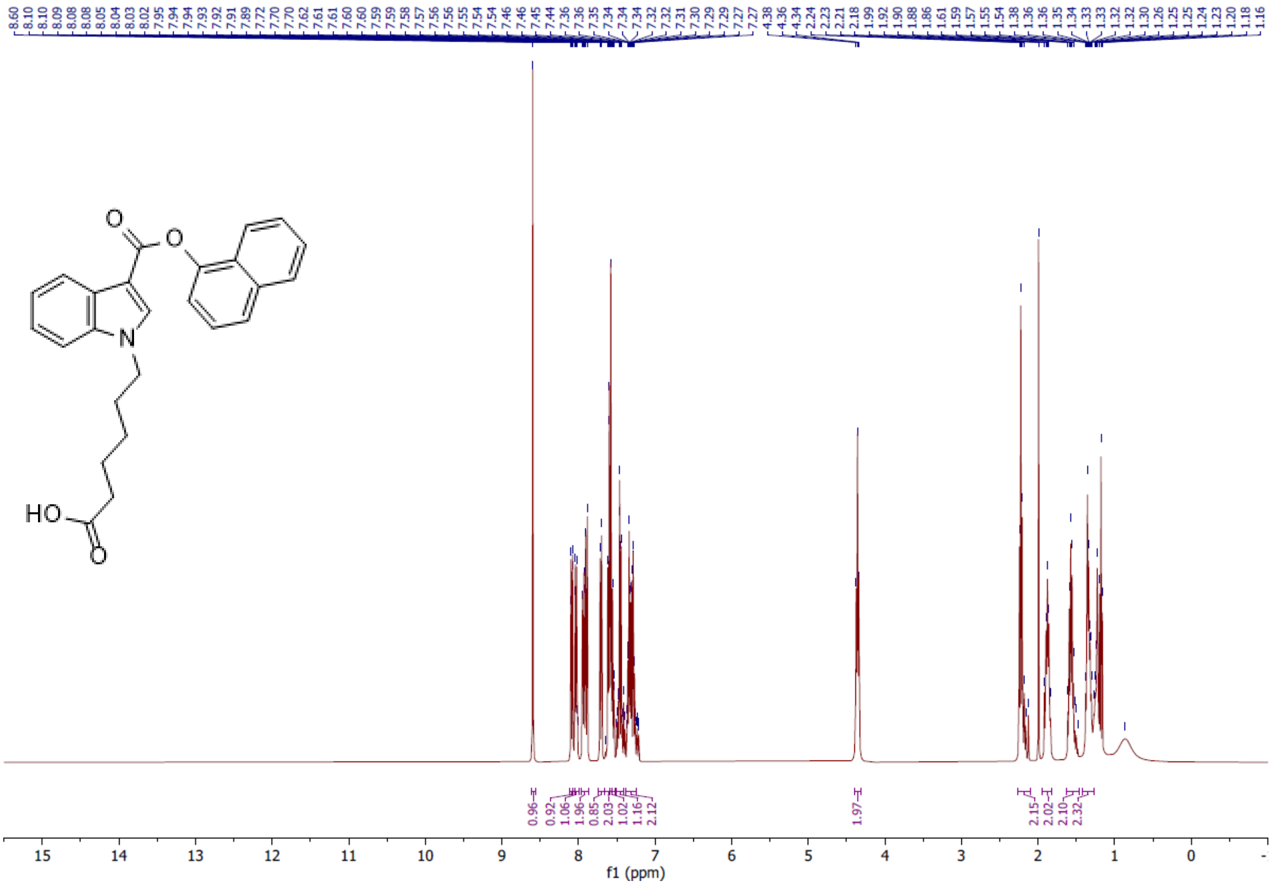

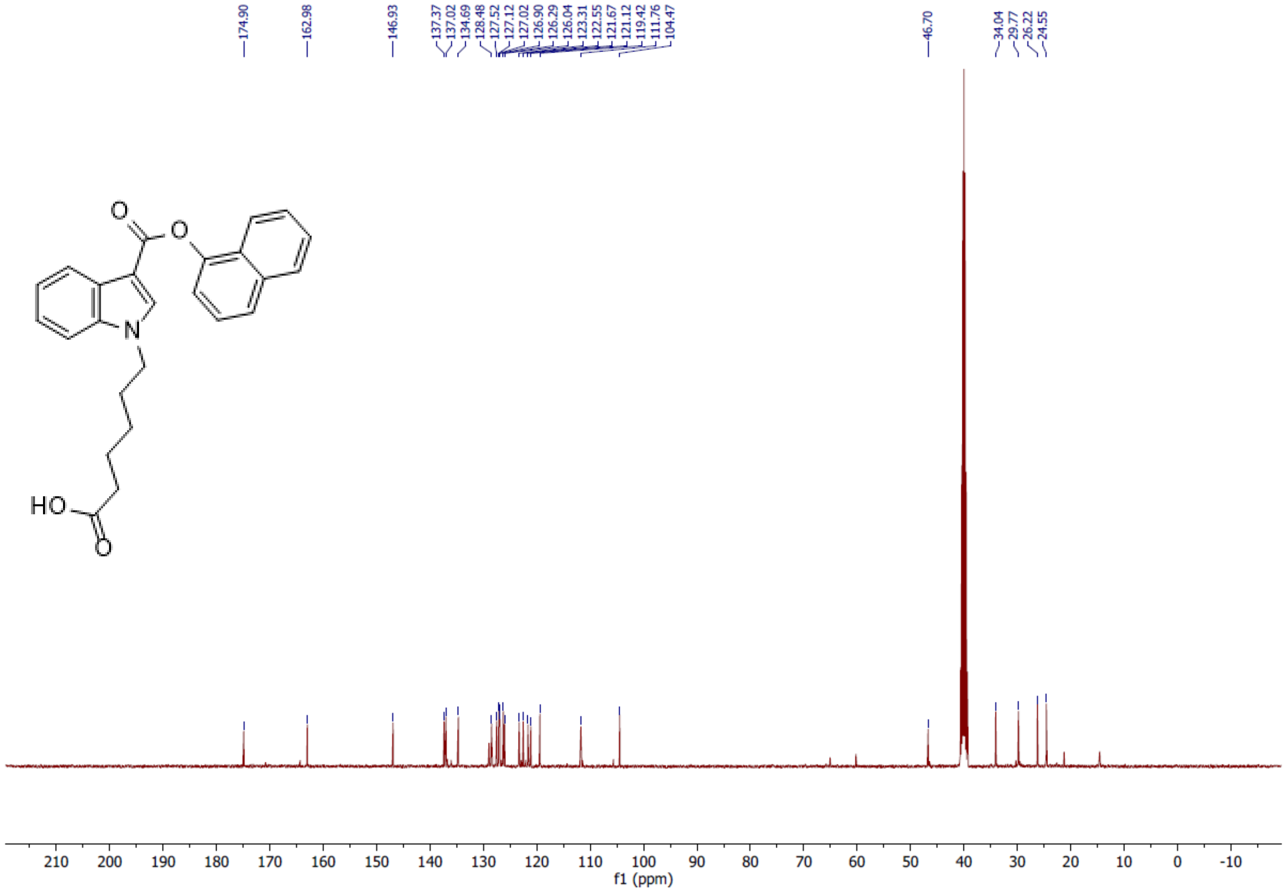
174.90
162.98
146.93
137.37
137.02
134.69
128.48
127.52
127.12
127.02
126.90
126.29
126.04
123.31
122.55
121.67
121.12
119.42
111.76
104.47
46.70
34.04
29.77
26.22
24.55
O
O
N
HO
O
210 200 190 180 170 160 150 140 130 120 110 100 90 80 70 60 50 40 30 20 10 0 -10
f1 (ppm)

Hapten 3

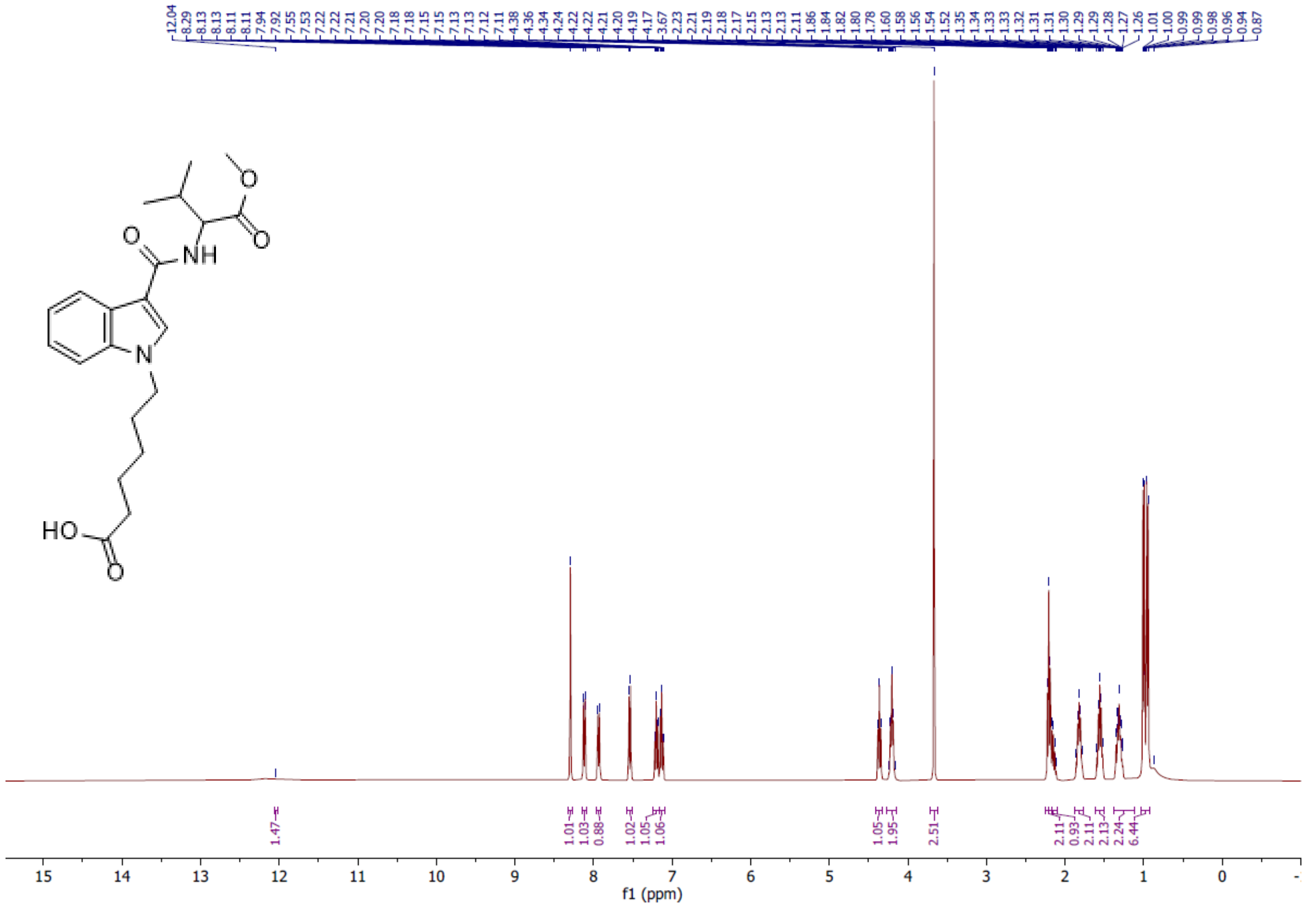
12.04
8.29
8.13
8.13
8.11
8.11
7.94
7.92
7.55
7.53
7.22
7.22
7.21
7.20
7.20
7.18
7.18
7.15
7.15
7.13
7.13
7.12
7.11
4.38
4.36
4.34
4.24
4.22
4.22
4.21
4.20
4.19
4.17
3.67
2.23
2.21
2.19
2.18
2.17
2.15
2.13
2.13
2.11
1.86
1.84
1.82
1.80
1.78
1.60
1.58
1.56
1.54
1.52
1.35
1.34
1.33
1.33
1.32
1.31
1.31
1.30
1.29
1.29
1.28
1.27
1.26
1.01
1.00
0.99
0.99
0.98
0.96
0.94
0.87
1.47
1.01
1.03
0.88
1.02
1.05
1.06
1.05
1.95
2.51
2.11
0.93
2.11
2.13
2.24
6.44
f1 (ppm)

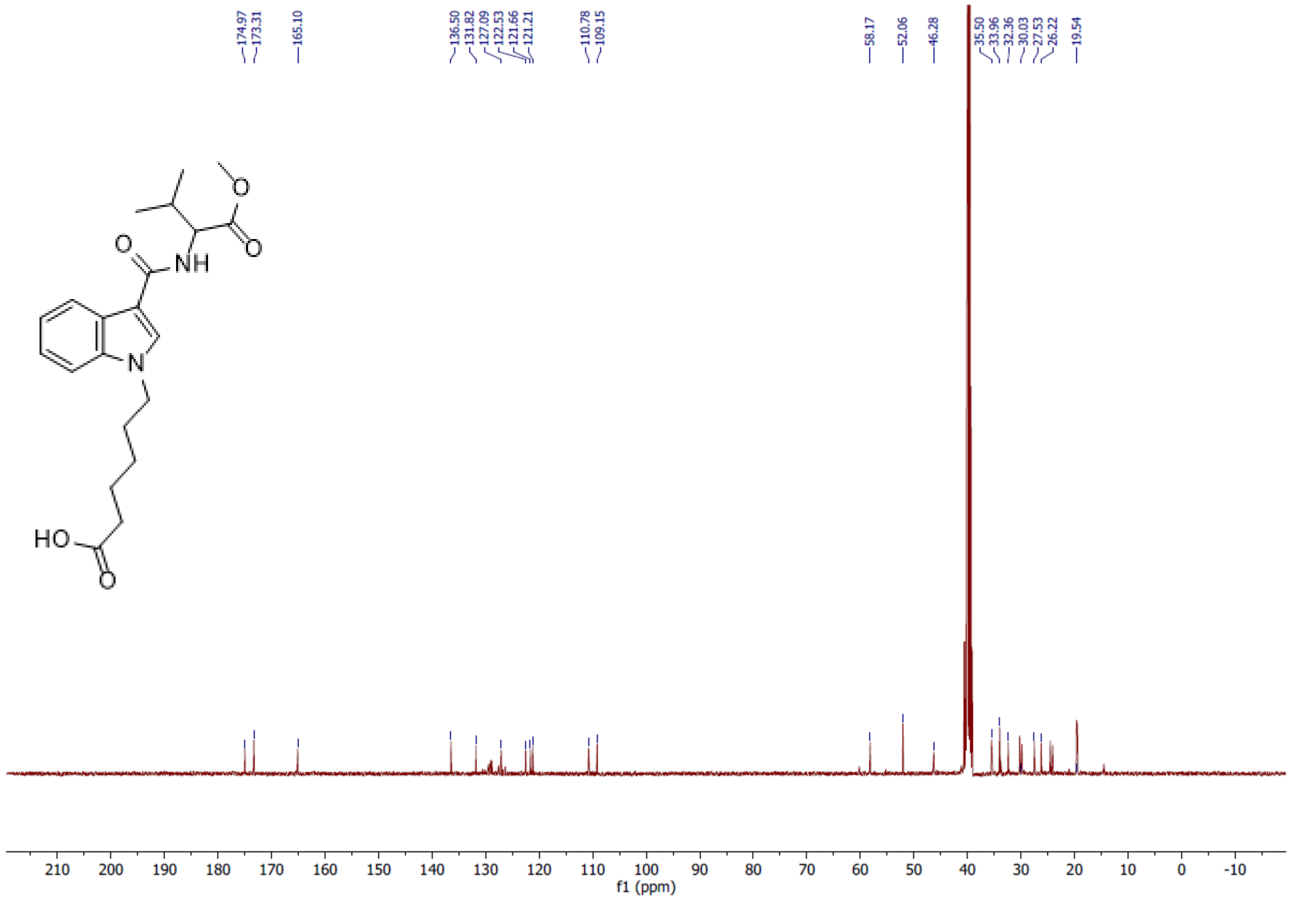
174.97
173.31
165.10
136.50
131.82
127.09
122.53
121.66
121.21
110.78
109.15
58.17
52.06
46.28
35.50
33.96
32.36
30.03
27.53
26.22
19.54
f1 (ppm)

Hapten 4

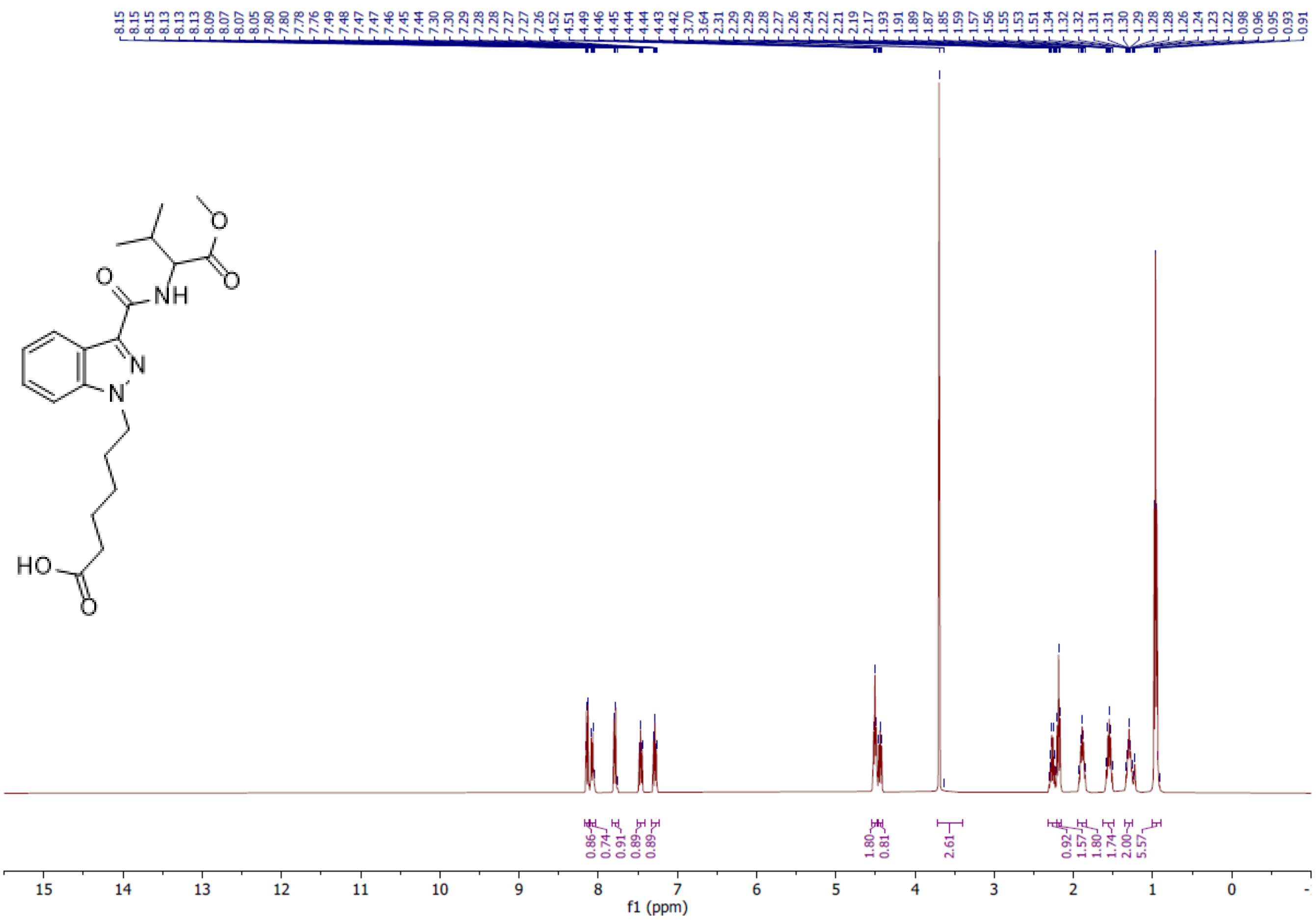

NMR Spectra – SCRAs:

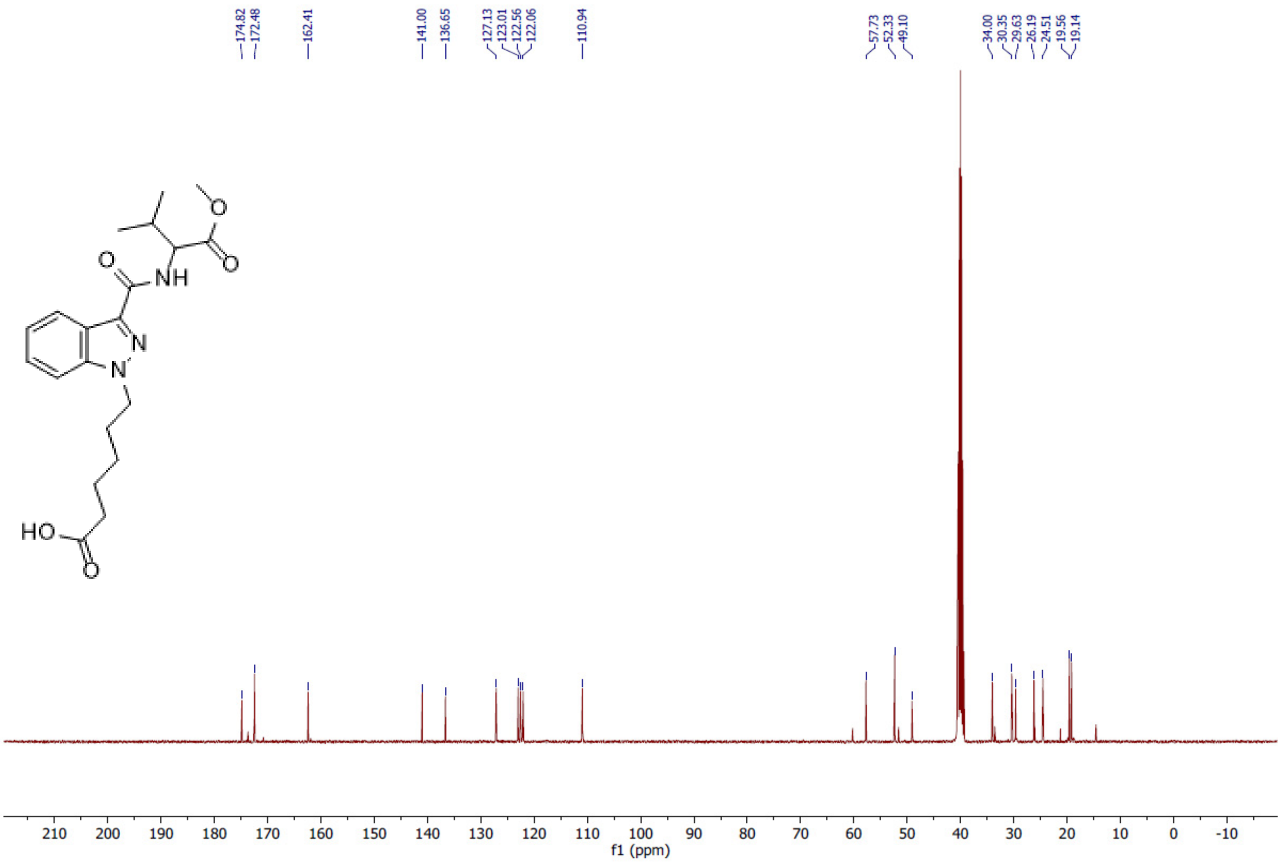

JWH-018 (**1**)

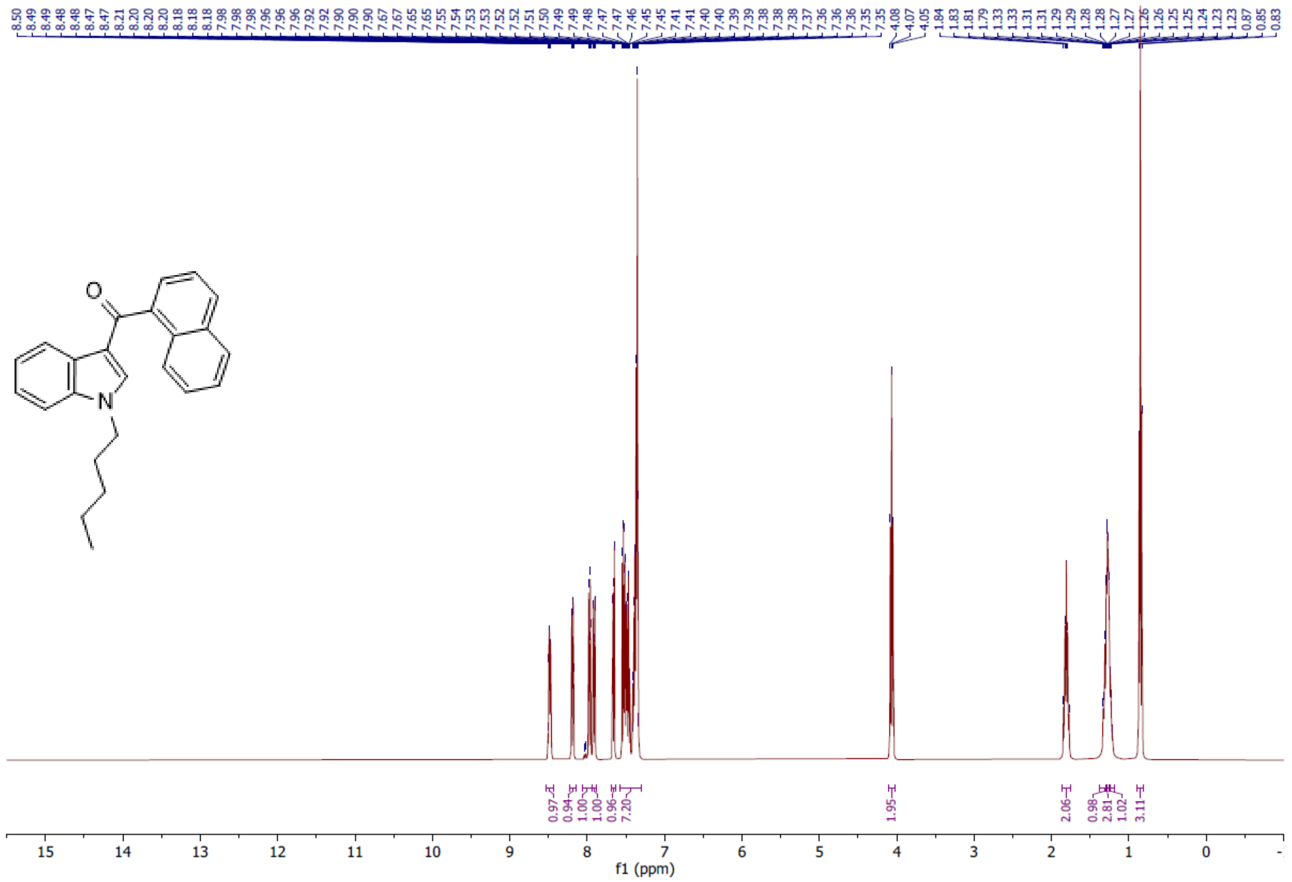

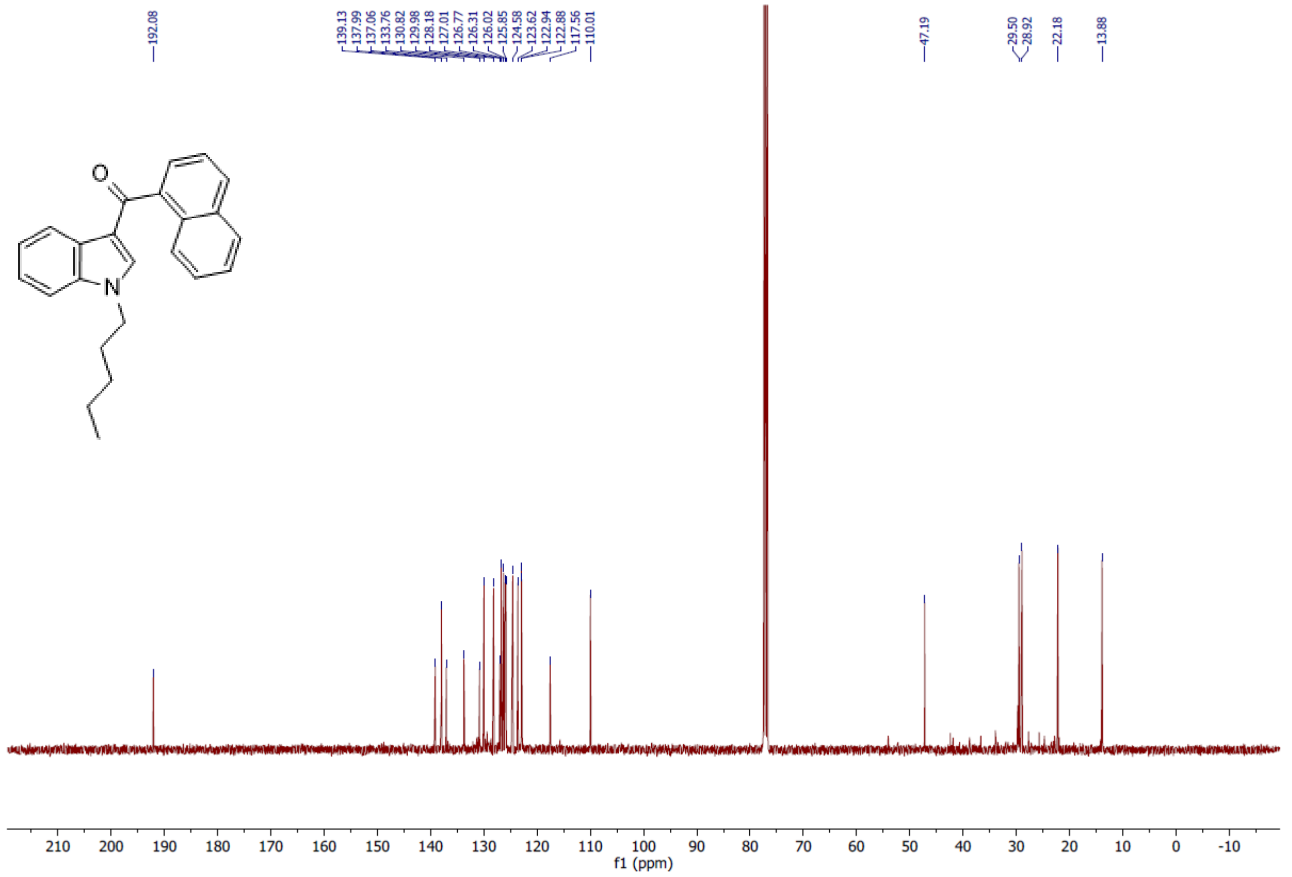

AM-2201 (**2**)

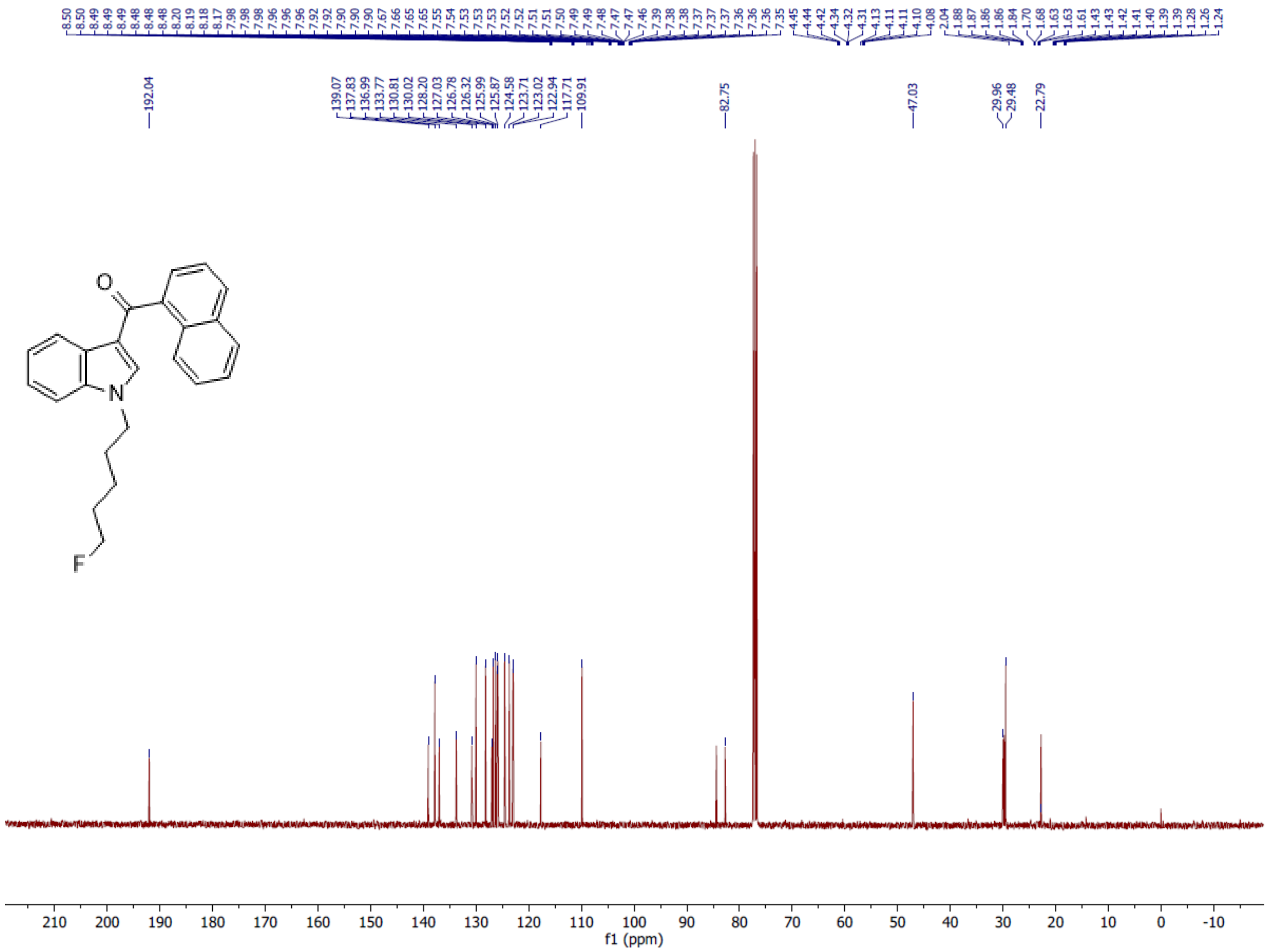

8.50 8.50 8.49 8.49 8.49 8.48 8.48 8.48 8.20 8.19 8.18 8.17 7.98 7.98 7.98 7.96 7.96 7.96 7.92 7.92 7.90 7.90 7.90 7.67 7.66 7.65 7.65 7.55 7.54 7.53 7.53 7.53 7.52 7.52 7.51 7.51 7.50 7.49 7.49 7.48 7.47 7.47 7.46 7.39 7.38 7.38 7.37 7.37 7.37 7.36 7.36 7.36 7.35 4.45 4.44 4.42 4.34 4.32 4.31 4.13 4.13 4.11 4.11 4.10 4.08 2.04 1.88 1.87 1.86 1.86 1.84 1.70 1.68 1.63 1.63 1.61 1.43 1.43 1.42 1.41 1.40 1.39 1.39 1.28 1.26 1.24
192.04
139.07 137.83 136.99 133.77 130.81 130.02 128.20 127.03 126.78 126.32 125.99 125.87 124.58 123.71 123.02 122.94 117.71 109.91
82.75
47.03
29.96 29.48
22.79
O
N
F
210 200 190 180 170 160 150 140 130 120 110 100 90 80 70 60 50 40 30 20 10 0 -10
f1 (ppm)

MMB-PICA (**3**)

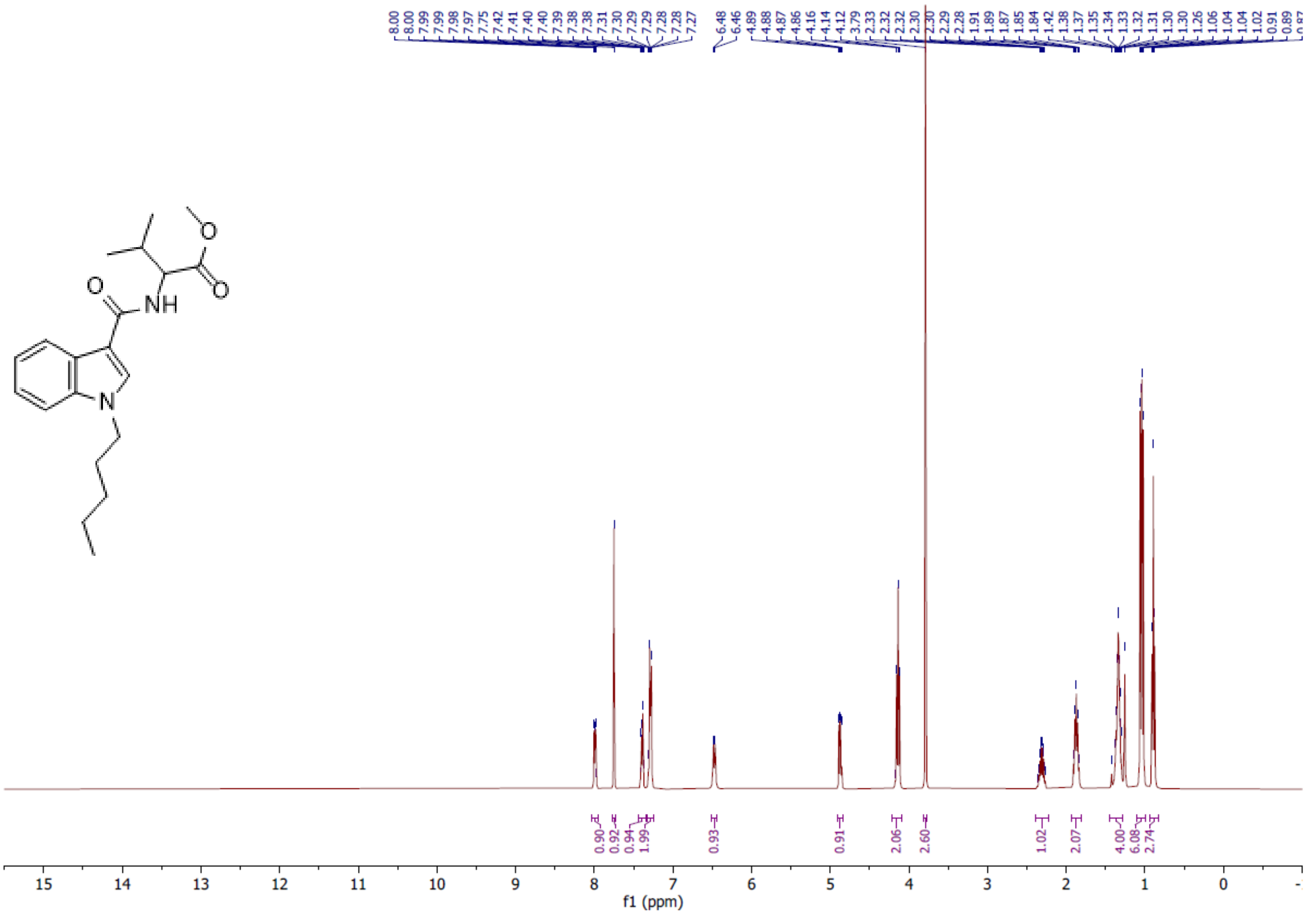
8.00
8.00
7.99
7.98
7.97
7.75
7.42
7.41
7.40
7.40
7.39
7.38
7.38
7.31
7.30
7.29
7.29
7.28
7.28
7.27
6.48
6.46
4.89
4.88
4.87
4.86
4.16
4.14
4.12
3.79
2.33
2.32
2.32
2.30
2.30
2.29
2.28
1.91
1.89
1.87
1.85
1.84
1.42
1.38
1.37
1.35
1.34
1.33
1.32
1.31
1.30
1.30
1.26
1.06
1.04
1.04
1.02
0.91
0.89
O
NH
O
N
O
0.90
0.92
0.94
1.99
0.93
0.91
2.06
2.60
1.02
2.07
4.00
6.08
2.74
15 14 13 12 11 10 9 8 7 6 5 4 3 2 1 0
f1 (ppm)

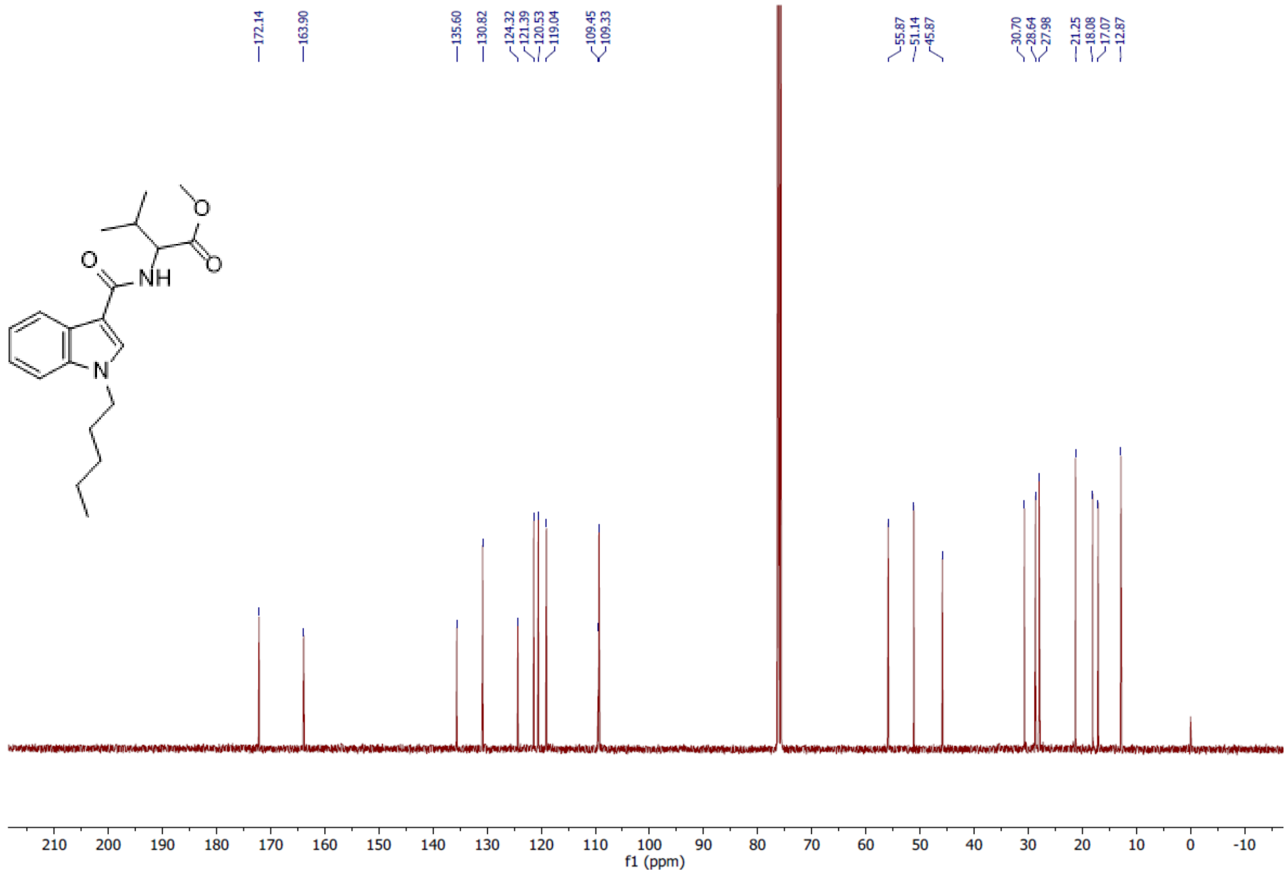
172.14
163.90
135.60
130.82
124.32
121.39
120.53
119.04
109.45
109.33
55.87
51.14
45.87
30.70
28.64
27.98
21.25
18.08
17.07
12.87
O
NH
O
N
O
210 200 190 180 170 160 150 140 130 120 110 100 90 80 70 60 50 40 30 20 10 0 -10
f1 (ppm)

5F-MMB-PICA (**4**)

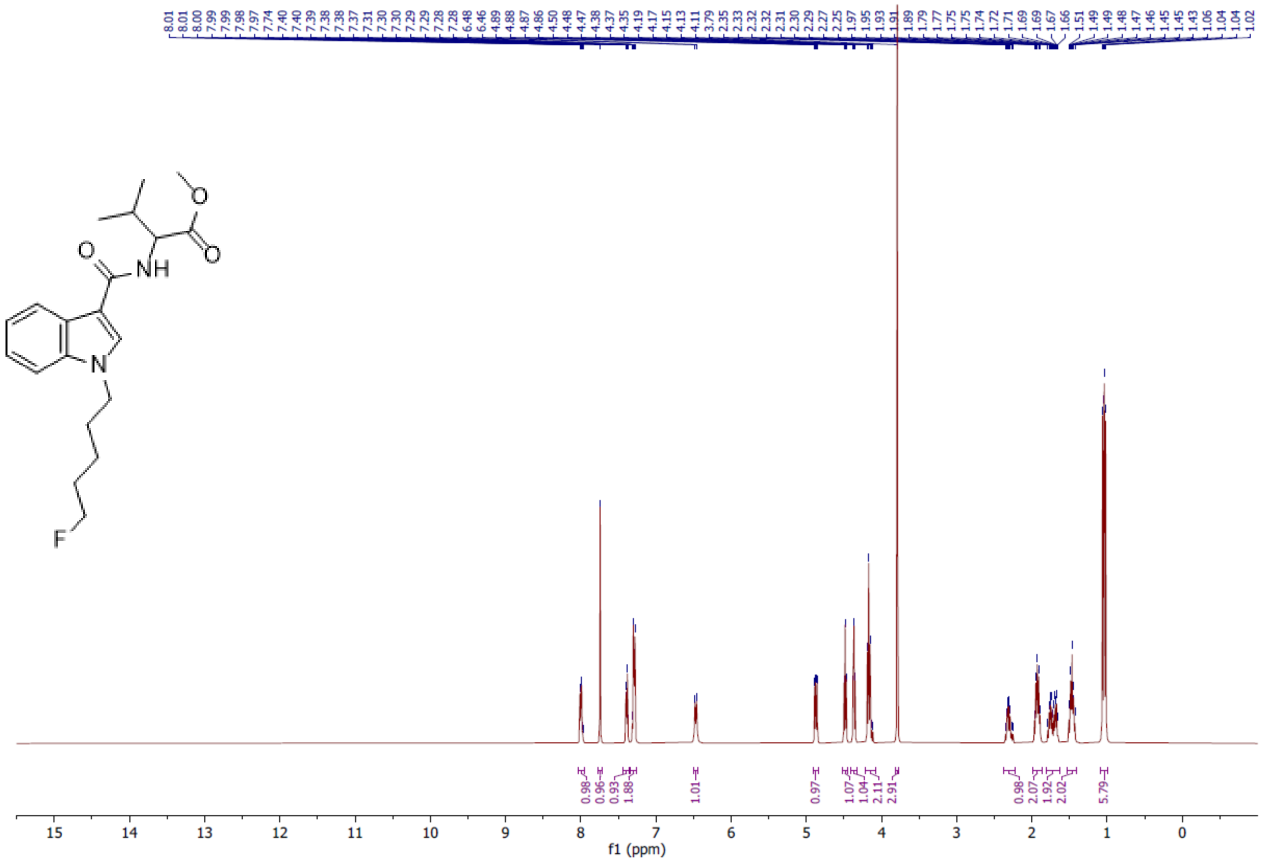
8.01 8.01 8.00 7.99 7.99 7.98 7.97 7.74 7.40 7.39 7.38 7.38 7.37 7.31 7.30 7.30 7.29 7.29 7.28 7.28 6.48 6.46 4.89 4.88 4.87 4.86 4.50 4.48 4.47 4.38 4.37 4.35 4.19 4.17 4.15 4.13 4.11 3.79 2.35 2.33 2.32 2.32 2.31 2.30 2.29 2.27 2.25 1.97 1.95 1.93 1.91 1.89 1.79 1.77 1.75 1.75 1.74 1.72 1.71 1.69 1.69 1.67 1.66 1.51 1.49 1.49 1.48 1.47 1.46 1.45 1.45 1.43 1.06 1.04 1.04 1.02
O
NH
O
O
N
F
0.98 0.96 0.93 1.88 1.01 0.97 1.07 1.04 2.11 2.91 0.98 2.07 1.92 2.02 5.79
15 14 13 12 11 10 9 8 7 6 5 4 3 2 1 0
f1 (ppm)

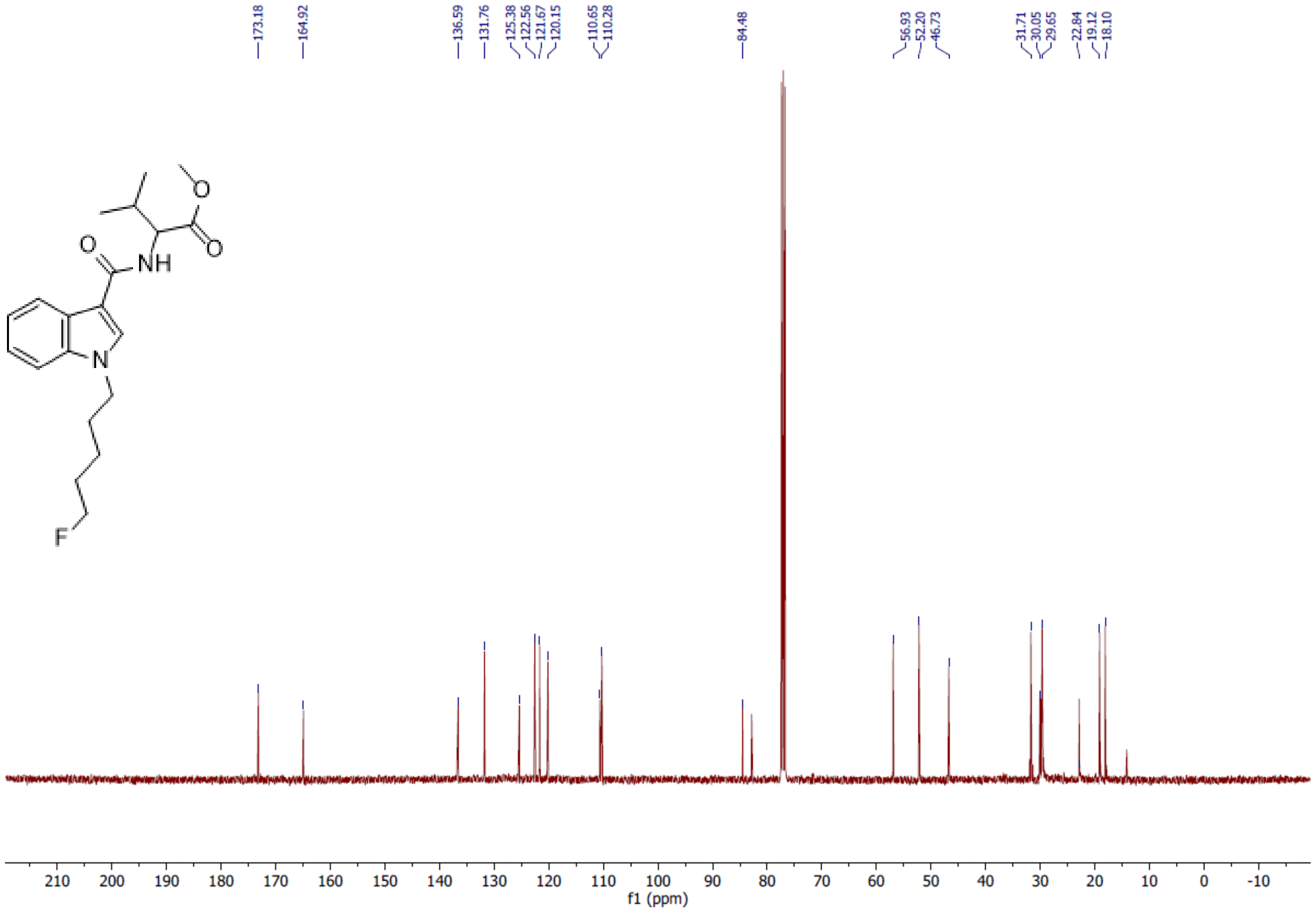
173.18
164.92
136.59
131.76
125.38
122.56
121.67
120.15
110.65
110.28
84.48
56.93
52.20
46.73
31.71
30.05
29.65
22.84
19.12
18.10
O
O
O
NH
N
F
210 200 190 180 170 160 150 140 130 120 110 100 90 80 70 60 50 40 30 20 10 0 -10
f1 (ppm)

MMB-PINACA (**5**)

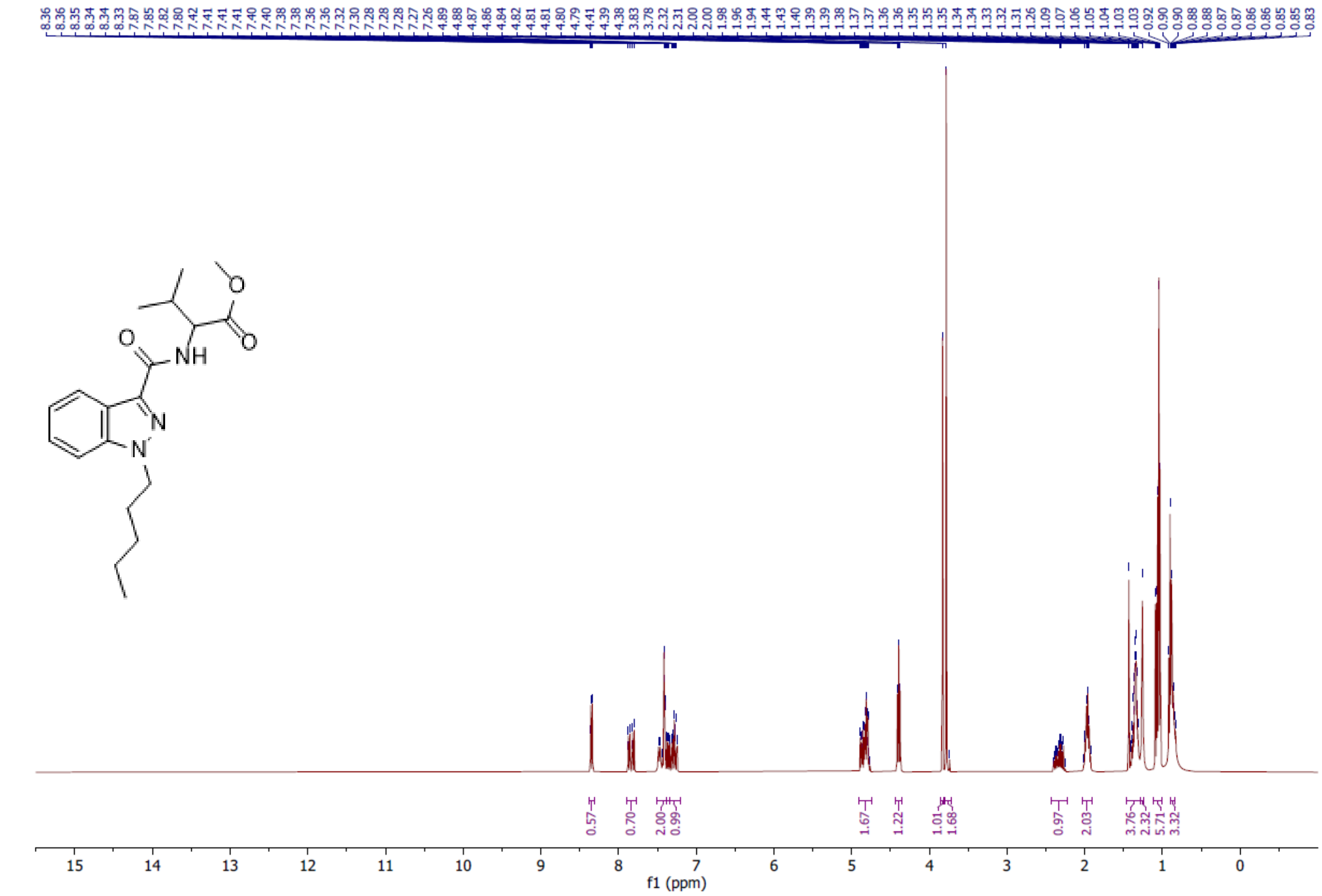

8.36 8.36 8.35 8.34 8.34 8.33 7.87 7.85 7.82 7.80 7.42 7.41 7.41 7.41 7.40 7.38 7.38 7.36 7.36 7.32 7.30 7.28 7.28 7.28 7.27 7.26 4.89 4.88 4.87 4.86 4.84 4.82 4.81 4.81 4.80 4.79 4.41 4.39 4.38 3.83 3.78 2.32 2.31 2.00 2.00 1.98 1.96 1.94 1.44 1.43 1.40 1.39 1.39 1.38 1.37 1.37 1.36 1.36 1.35 1.35 1.35 1.34 1.34 1.33 1.33 1.32 1.31 1.26 1.09 1.07 1.06 1.05 1.04 1.03 1.03 0.92 0.90 0.90 0.88 0.88 0.87 0.87 0.86 0.86 0.85 0.85 0.83
O
NH
O
O
N
N
0.57
0.70
2.00
0.99
1.67
1.22
1.01
1.68
0.97
2.03
3.76
2.32
5.71
3.32
15 14 13 12 11 10 9 8 7 6 5 4 3 2 1 0
f1 (ppm)

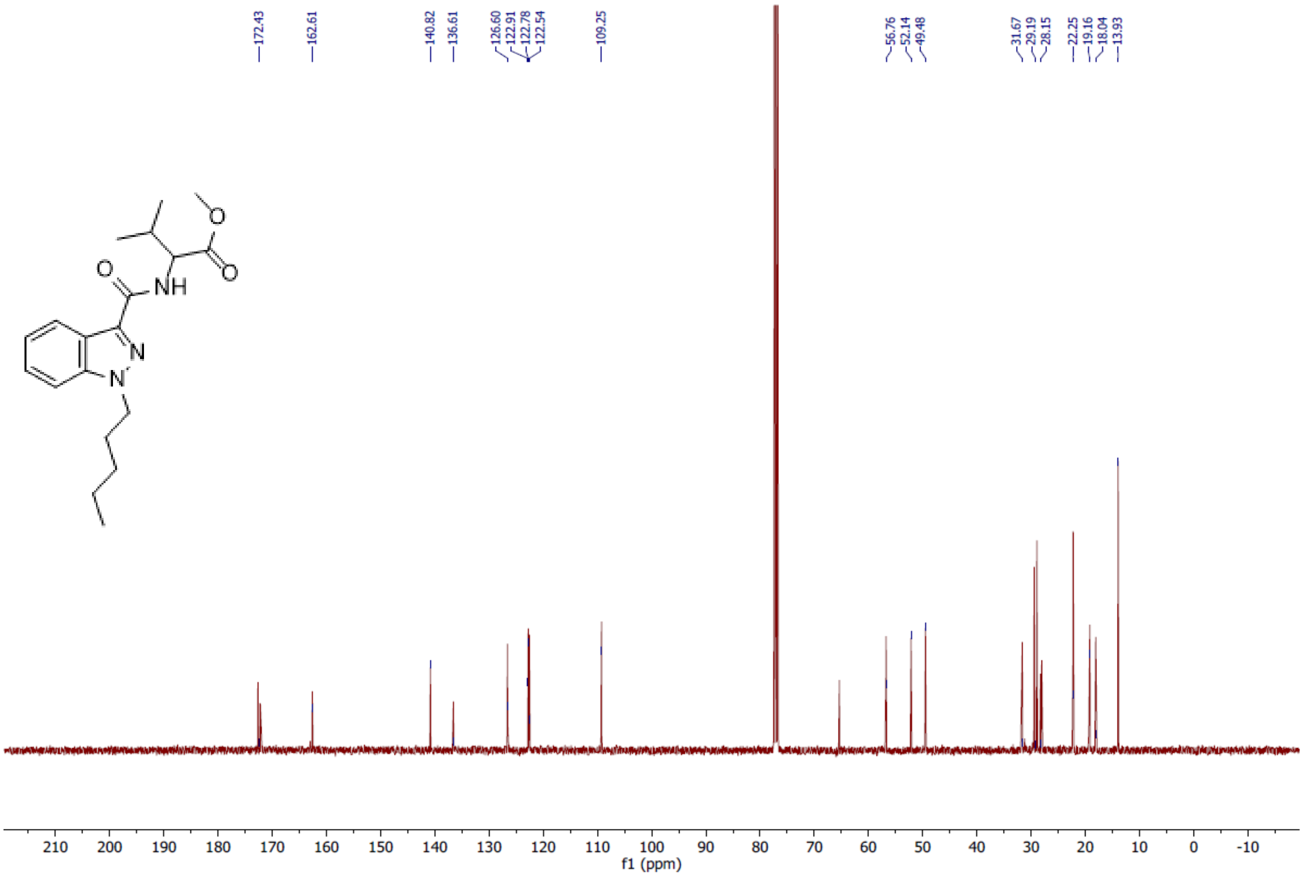

172.43
162.61
140.82
136.61
126.60
122.91
122.78
122.54
109.25
56.76
52.14
49.48
31.67
29.19
28.15
22.25
19.16
18.04
13.93
O
NH
O
O
N
N
210 200 190 180 170 160 150 140 130 120 110 100 90 80 70 60 50 40 30 20 10 0 -10
f1 (ppm)

5F-MMB-PINACA (**6**)

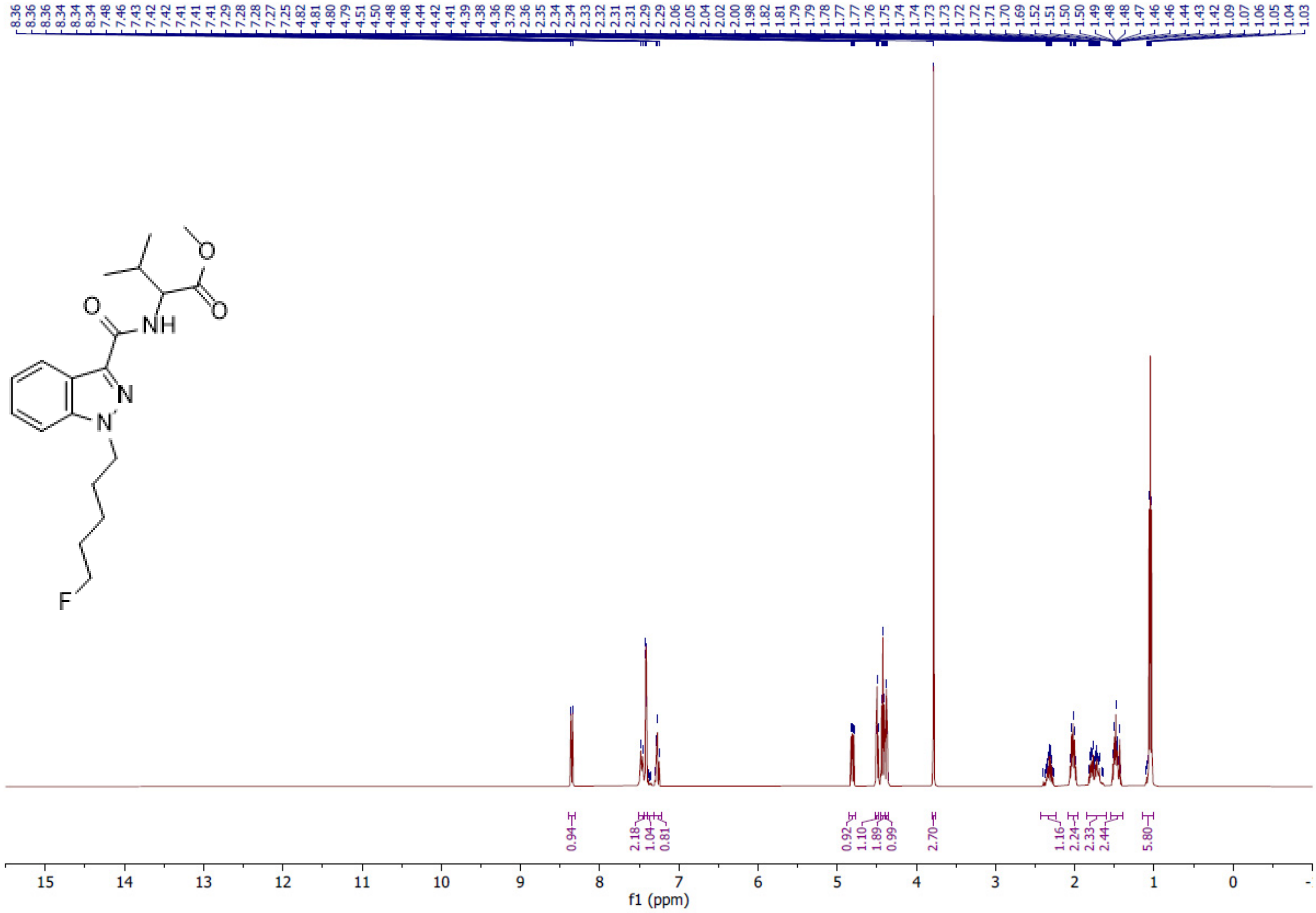

AMB-FUBINACA (**7**)

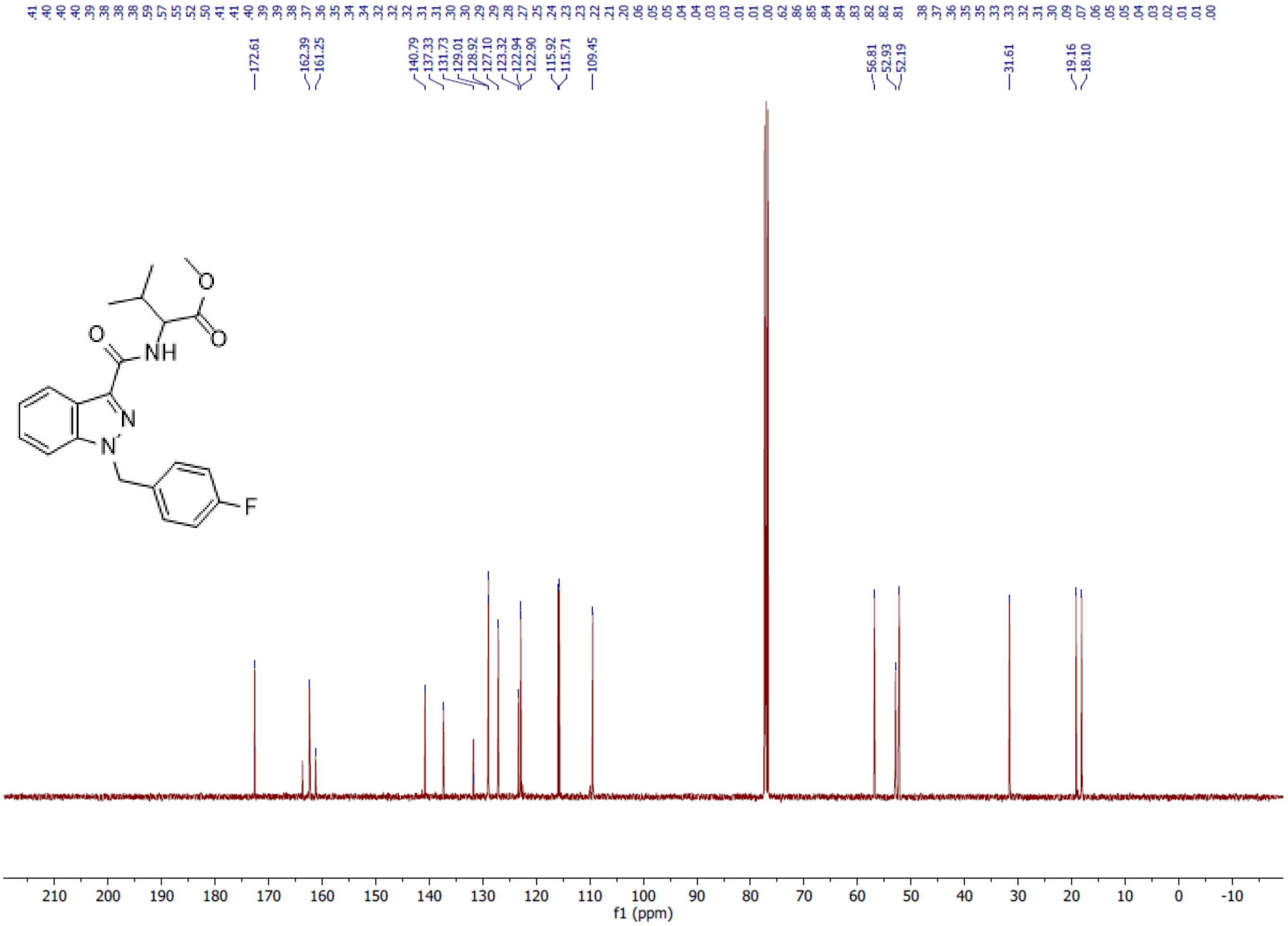

NAPIC (**8**)

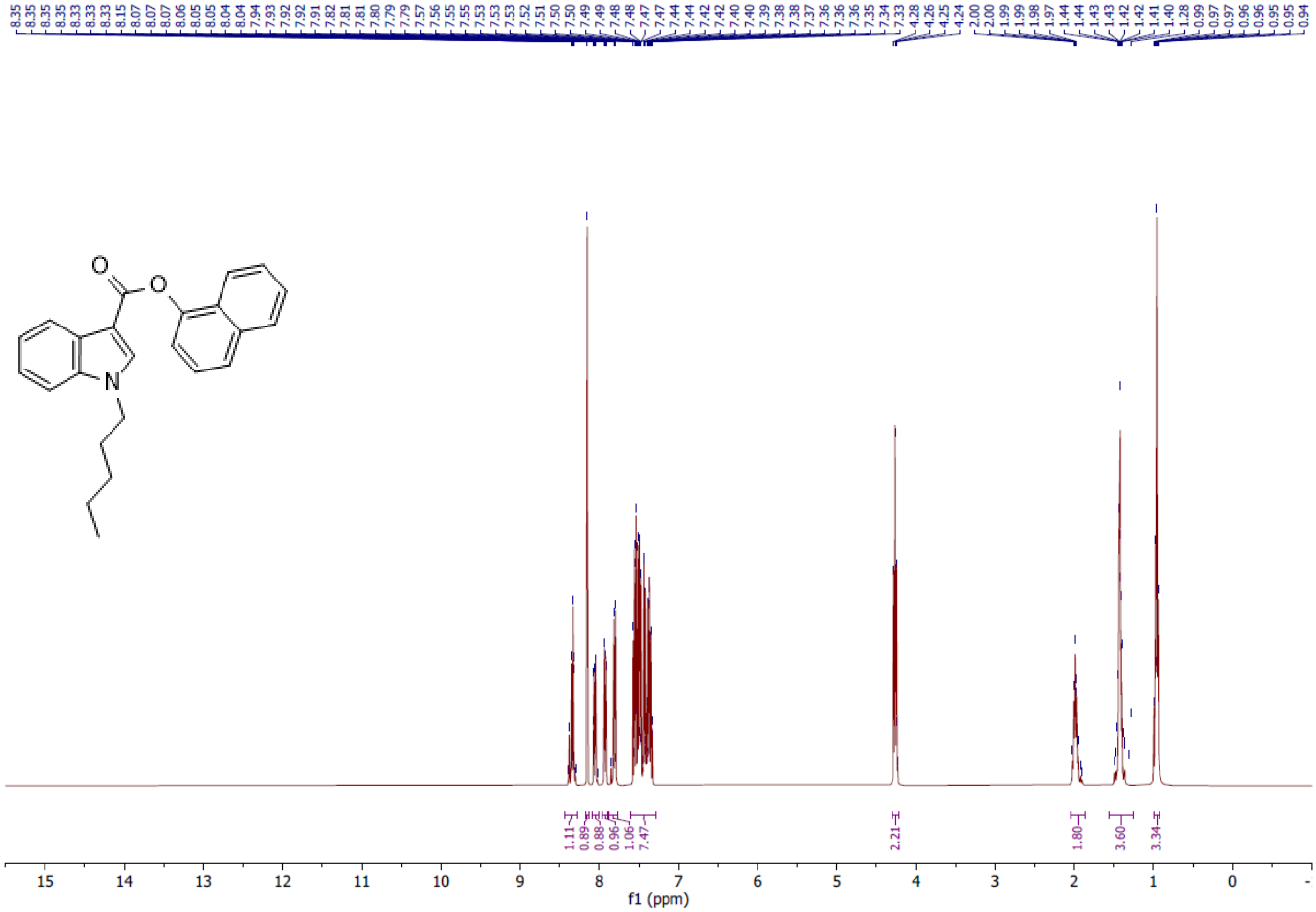
8.35 8.35 8.35 8.33 8.33 8.33 8.15 8.07 8.07 8.06 8.05 8.05 8.04 8.04 7.94 7.93 7.92 7.92 7.91 7.82 7.81 7.81 7.80 7.79 7.79 7.57 7.56 7.55 7.55 7.53 7.53 7.53 7.52 7.51 7.50 7.49 7.49 7.48 7.48 7.47 7.47 7.44 7.44 7.42 7.42 7.40 7.40 7.39 7.38 7.38 7.37 7.36 7.36 7.36 7.35 7.34 7.33 4.28 4.26 4.25 4.24 2.00 2.00 1.99 1.99 1.98 1.97 1.44 1.44 1.43 1.43 1.42 1.42 1.41 1.40 1.28 0.99 0.97 0.97 0.96 0.96 0.95 0.95 0.94
1.11 0.89 0.88 0.96 1.06 7.47 2.21 1.80 3.60 3.34
O
O
N
15 14 13 12 11 10 9 8 7 6 5 4 3 2 1 0
f1 (ppm)

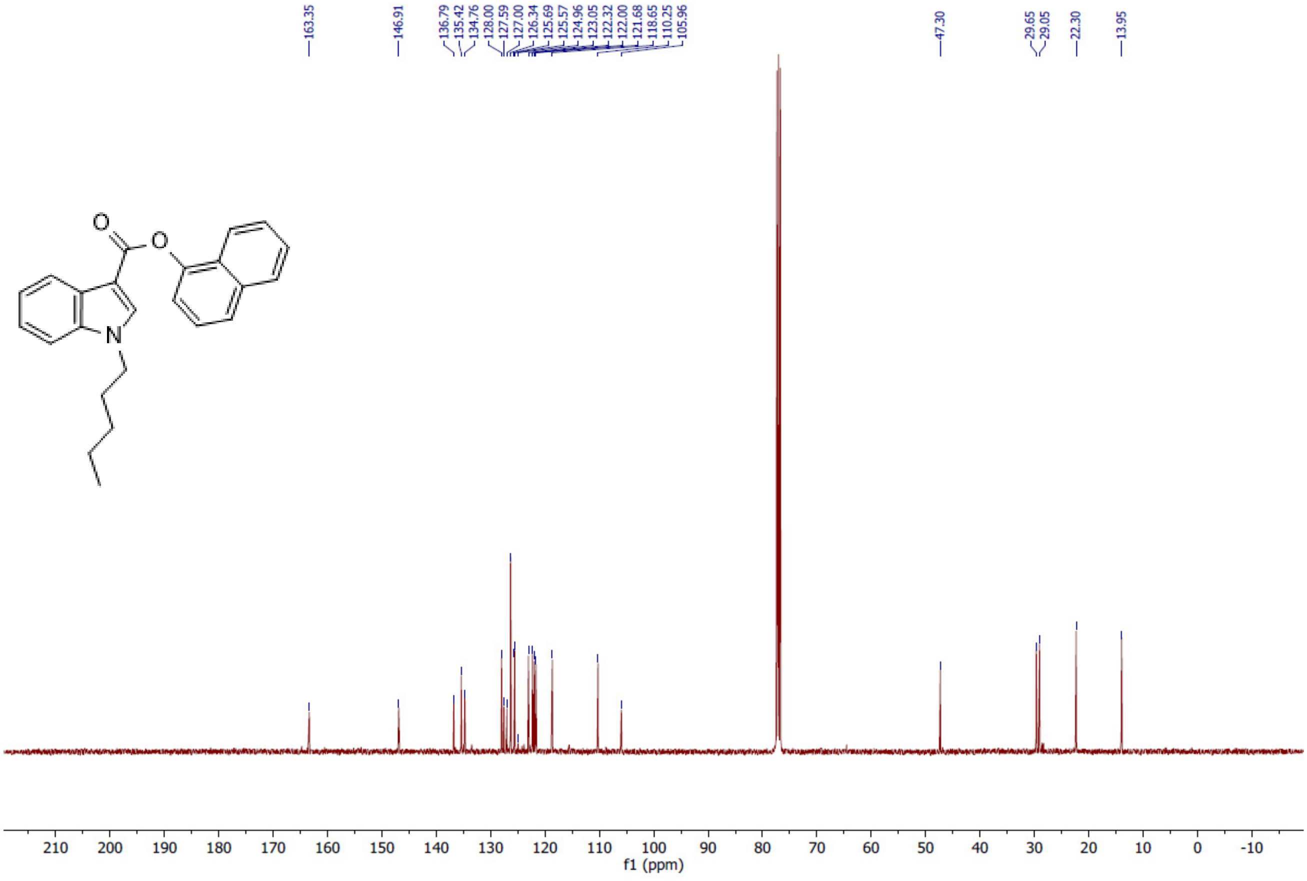
163.35
146.91
136.79 135.42 134.76 128.00 127.59 127.00 126.34 125.69 125.57 124.96 123.05 122.32 122.00 121.68 118.65 110.25 105.96
47.30
29.65 29.05
22.30
13.95
O
O
N
210 200 190 180 170 160 150 140 130 120 110 100 90 80 70 60 50 40 30 20 10 0 -10
f1 (ppm)
